# PRKCSH deficiency promotes an anti-tumor immune microenvironment via UPR activation and M1 macrophage polarization

**DOI:** 10.1186/s12935-025-04104-2

**Published:** 2025-12-05

**Authors:** Guo Xiyuan, Worapong Khaodee, Yang Jianghua, Xiaoke Sun, Piyawan Bunpo, Yuan Yulin, Yuan Qing, Ratchada Cressey

**Affiliations:** 1 Department of Medical Technology, Faculty of Associated Medical Sciences, Chiang Mai University, Chiang Mai, Thailand; 2 Public Center of Experimental Technology, The School of Basic Medical Sciences, Southwest Medical University, Luzhou, China; 3 Cancer Research Unit, Department of Medical Technology, Faculty of Associated Medical Sciences, Chiang Mai University, Chiang Mai, Thailand; 4 The Affiliated Stomatological Hospital, Southwest Medical University, Luzhou, 646000 China; 5 Key Laboratory of Medical Electrophysiology of the Ministry of Education, Medical Electrophysiological Key Laboratory of Sichuan Province, Institute of Cardiovascular Research, Southwest Medical University, Luzhou, 646000 China

**Keywords:** PRKCSH, Glucosidase II beta subunit, IRE1α, ER stress, Macrophage polarization, Tumor microenvironment

## Abstract

Lung adenocarcinoma remains one of the most common causes of cancer deaths. The tumor grows by avoiding the immune system and adapting to stress in the endoplasmic reticulum. The IRE1α–XBP1 pathway is a key pathway for cells to sense stress in the endoplasmic reticulum and has a large effect on the immune system. *PRKCSH* encodes a regulatory subunit of glucosidase II that helps keep the endoplasmic reticulum in balance by modifying how IRE1α works. However, it is unclear how it affects tumor immunity. This study used clinical sample analysis, bioinformatic analysis, CRISPR/Cas9-mediated gene deletion, cytokine profiling, macrophage co-culture, and zebrafish xenograft experiments to investigate the immunological role of *PRKCSH*. *PRKCSH* deficiency reduced basal IRE1α phosphorylation but led to exaggerated activation under ER stress, including increased XBP1s and p-JNK signaling. IL-6 and IL-8 secretion was suppressed in *PRKCSH*-knockout (KO) cancer cells, disrupting cytokine-mediated immune suppression. Conditioned media from *PRKCSH*-KO cells enhanced M1 macrophage polarization in vitro, as evidenced by increased CD86⁺ macrophages and expression of key M1-polarization markers. These effects were corroborated in zebrafish xenografts, where *PRKCSH* deficiency diverted the immune environment toward an M1-dominant phenotype. Analysis of clinical pleural effusion samples further validated these findings, revealing a significantly reduced M1/M2 macrophage ratio in malignant versus benign conditions. Furthermore, *PRKCSH*-KO cells exhibited increased susceptibility to ER stress-induced apoptosis and ferroptosis, along with impaired autophagy. In conclusion, our findings place *PRKCSH* as a key regulator linking ER stress signaling with tumor immune evasion and cell death pathways. Targeting *PRKCSH* may represent a promising therapeutic strategy to promote ferroptosis and anti-tumor immunity in lung adenocarcinoma.

**Supplementary Information:**

The online version contains supplementary material available at 10.1186/s12935-025-04104-2.

## Introduction

Lung cancer is still one of the most common causes of death from cancer, and the tumor microenvironment (TME) is mostly what makes tumors grow and avoid the immune system. Endoplasmic reticulum (ER) stress is a major part of the TME. It happens when there isn’t enough oxygen, nutrients, or oxidative stress. To survive these hostile conditions, cancer cells activate the unfolded protein response (UPR), a homeostatic mechanism that initially promotes survival but can also contribute to immune modulation and tumor progression under chronic stress [1, 2]. The IRE1α/XBP1 axis is especially important for both tumor survival and immune regulation among the three UPR branches. When IRE1α is turned on, it starts its RNase function, which causes XBP1 mRNA splicing. This affects the secretion of cytokines, the ability to avoid the immune system, and the ability to adapt to changes in metabolism [3, 4]. However, IRE1α signaling depends on the situation. It can help tumors grow and suppress the immune system, but it can also help the immune system fight tumors in some situations [5–7].

PRKCSH (protein kinase C substrate 80 K-H) is a non-catalytic part of glucosidase II that helps control IRE1α activity. It helps IRE1α autophosphorylation and oligomerization, which makes its RNase function stronger [8]. In lung adenocarcinoma, high levels of PRKCSH expression have been linked to a poor prognosis, immune suppression, and resistance to cell death caused by stress [8–15]. However, it’s still not clear if PRKCSH helps the immune system avoid detection by changing how ER stress signals and immune cells work in the TME. In cancer, macrophage polarization is an important part of immune regulation. M1 macrophages help the body fight inflammation and cancer, while M2 macrophages lower immunity and help tumors grow [16]. Researchers have shown that IRE1α signaling affects how macrophages act [17], but it is not clear if PRKCSH is involved in this process.

Moreover, IRE1α has been linked to the regulation of key cytokines such as IL-6 and IL-8—both central to immune recruitment, angiogenesis, and tumor-associated inflammation [18–20]. Whether PRKCSH indirectly affects cytokine profiles through its interaction with IRE1α is yet to be determined. In this study, we investigated whether PRKCSH affects the immune system and the stress responses of the endoplasmic reticulum (ER) in lung adenocarcinoma. We focus on its possible role in macrophage polarization and cytokine regulation.

## Methods

### Collection of clinical specimens and isolation of cells

Fresh tumor tissues and paired adjacent normal tissues (sampled at a distance > 5 cm from the tumor margin) were obtained from treatment-naïve patients with histologically confirmed primary non-small cell lung cancer (NSCLC) undergoing surgical resection at the Affiliated Hospital of Southwest Medical University. In addition, pleural effusion samples were collected from patients diagnosed with either benign pleural effusion (BPE) or malignant pleural effusion (MPE) for comparative immune phenotyping. Human peripheral blood mononuclear cells (PBMCs) from healthy donors were also collected for use in co-culture experiments. All samples were obtained under protocols approved by the Clinical Trial Ethics Committee of the Affiliated Hospital of Southwest Medical University (Permission no. KY2023277), with informed consent obtained from all participants prior to collection.

Primary tumor and adjacent normal tissues were snap-frozen in liquid nitrogen immediately following surgical resection for downstream molecular analysis. These samples were subsequently used for Western blot analysis to identify tumor-specific and adjacent tissue-specific alterations. Pleural effusion samples were processed for flow cytometry to assess macrophage polarization profiles in clinical contexts of malignancy versus benignity.

### Bioinformatic analysis of PRKCSH impact on the immune landscape of lung adenocarcinoma

We used a variety of publicly available datasets and analytical platforms to do an integrated bioinformatic analysis to investigate how PRKCSH expression might affect the immune system in lung adenocarcinoma (LUAD).

We looked at publicly available datasets to see how much PRKCSH was expressed in lung adenocarcinoma (LUAD) and normal lung tissues. We got immunohistochemical (IHC) staining data from the Human Protein Atlas (HPA) to check the expression of the PRKCSH protein. We also used gene expression data from the Gene Expression Omnibus (GEO) and The Cancer Genome Atlas (TCGA) to look at the differences in PRKCSH transcript levels between tumor and normal tissues. We used the Wilcoxon rank-sum test to find out if the differences in expression were statistically significant.

Using Gene Set Enrichment Analysis (GSEA) to Find Pathways: We used transcriptomic data from the TCGA-LUAD dataset to do Gene Set Enrichment Analysis (GSEA) to find the biological pathways that are linked to PRKCSH overexpression. We used hallmark gene sets from the Molecular Signatures Database (MSigDB) for enrichment analysis [21]. To find out if something was statistically significant, we calculated the normalized enrichment scores (NES) and the false discovery rate (FDR). We thought that pathways with an FDR of less than 0.05 were significantly enriched.

To explore the potential immunomodulatory role of PRKCSH, Spearman correlation analysis was performed between PRKCSH expression and immune-related genes in the TCGA-LUAD dataset. This included cytokines (IL6, TGFB1), immune checkpoints (PDCD1, LAG3), and co-stimulatory molecules (CD86, CD80). We calculated a correlation coefficient (r) and the p-values that went with it. A correlation was considered significant if *p* < 0.05.

Using CIBERSORT to look at immune cell infiltration: CIBERSORT is a deconvolution algorithm that estimates immune cell fractions from bulk gene expression data [22]. It was used to look at the link between PRKCSH expression and immune cell composition. We used CIBERSORT on the TCGA-LUAD dataset to measure how much more of certain immune cell types, like T lymphocytes, B lymphocytes, natural killer (NK) cells, macrophages, neutrophils, eosinophils, and mast cells there were. We used the Wilcoxon rank-sum test to compare immune infiltration between groups with high and low PRKCSH expression. A p-value of less than 0.05 was considered significant.

We used the scCancerExplorer, an interactive web platform for pan-cancer single-cell transcriptomic analysis [23], to look at LUAD single-cell RNA-sequencing datasets (GSE127465, GSE117570, GSE162498) in more detail. This helped us understand PRKCSH expression at the single-cell level and how it relates to macrophage polarization. After checking the quality and normalizing the data, we used unsupervised clustering and Uniform Manifold Approximation and Projection (UMAP) to show the different groups of cells. Dot plots showed the expression of marker genes like PRKCSH, pan-macrophage marker CD68, M1 marker CD86, M2 marker CD163, and cytokines IL6, IL8, TGFB1, and TNF. The size of the dots in these plots shows the percentage of cells that are expressing the gene, and the color intensity shows the average expression. We used the platform’s default settings to run the analyses.

### Generation of knockout cells from lung adenocarcinoma cell line

PRKCSH-knockout (KO) A549 cells were generated using CRISPR-Cas9 technology as previously described [9]. A549 cells stably expressing Cas9 (A549-Cas9) were purchased from Ubigene Biosciences (Guangzhou, China) and transfected with a PRKCSH-targeting gRNA construct (YKO-LV001-hPRKCSH) via electroporation. A non-targeting gRNA control (YKO-LV001-Control, Ubigene Biosciences) was used as a negative control to validate the specificity of gene editing; this control group is referred to as WT-A549. After transfection, puromycin selection (2 µg/mL) was applied for 7 days. Surviving monoclonal populations were isolated using the limiting dilution method and verified by Western blot analysis. The identity and mycoplasma-free status of the parental A549 line were confirmed by the vendor (Ubigene Biosciences). Cells were routinely tested for mycoplasma contamination using PCR-based assays and cultured for no more than 20 passages to maintain consistency and reproducibility.

### Cell viability assay

PRKCSH-KO and control cells (3 × 10^3^) were seeded in 96-well plates at 40–50% confluence. After overnight incubation, cells were treated with 5 µg/mL tunicamycin to induce ER stress. Cell viability was measured at 0, 6, 12, 24, and 48 h using the CCK-8 assay by adding 10 µL of reagent and incubating for 4 h. Absorbance at 450 nm was read, and cell survival was calculated as: Survival rate (%) = ((A_sample - A_blank)/(A_control - A_blank)) × 100.

### Cell migration assay (Scratch Assay)

*PRKCSH*-KO and control cells were seeded in 96-well plates. After 24 h of incubation, scratches were made using a p200 pipette tip. Detached cells were removed by washing with PBS, and fresh medium with or without tunicamycin (5 µg/mL) was added. Images were captured at 0 and 72 h to evaluate scratch closure and cell migration.

### Apoptosis assay using Annexin V and 7-AAD

*PRKCSH*-KO and control cells were treated with 5 µg/mL tunicamycin for up to 48 h. Apoptosis was assessed using the Annexin V Apoptosis Detection Kit with 7-AAD. Cells were prepared, stained with Annexin V-APC and 7-AAD, and incubated for 15 min at room temperature, protected from light. Flow cytometry (BD Biosciences) was used to analyze early apoptosis (Annexin V^+^/7-AAD^−^) and late apoptosis/necrosis (Annexin V^+^/7-AAD^+^), providing a full apoptotic profile.

### Transmission electron microscopy

A549-WT cells and *PRKCSH*-KO A549 cells were subjected to TM-induced stress for 48 h. Cells were fixed in 2.5% glutaraldehyde solution (EMS) for 1 h at room temperature, followed by post-fixation with 1% osmium tetroxide (TED PELLA-US) for 1 h at room temperature. Samples were dehydrated in ascending concentrations of alcohol and acetone and embedded in Epon resin (SPI-US). Semithin Sect. (0.8 μm) were stained with toluidine blue, and ultrathin Sects. (600–900 Å) were prepared using a Leica ultracut microtome. Sections were mounted on uncoated copper grids and stained with saturated solutions of uranyl acetate and lead citrate. Images were acquired using a HITACHI HT7800 transmission electron microscope at an acceleration voltage of 80 kV.

### Western blot analysis

Protein expression levels of PRKCSH, cell death-related markers, ER stress markers, and proteins associated with the IRE1α-XBP1 signaling pathway were analyzed via Western blotting. Cells were lysed in RIPA buffer (1×, Santa Cruz Biotechnology) with protease/phosphatase inhibitors (1:100) for 30 min on ice. Protein concentrations were determined using the Enhanced BCA Protein Assay Kit (Beyotime Biotechnology). Equal protein amounts (25 µg) were separated on 10% SDS-PAGE gels and transferred to PVDF membranes. Membranes were blocked with 5% BSA, incubated overnight with primary antibodies at 4 °C, followed by HRP-conjugated secondary antibodies. Bands were visualized with chemiluminescence and quantified using ImageJ. The list of antibodies is available in supplementary Table 2.

### Determination of secreted cytokines

To measure secreted cytokines, levels of IL-6, IL-8 and VEGF in conditioned media from *PRKCSH*-KO and WT-A549 cells were quantified using a cytometric bead array (CBA), a bead-based immunoassay similar to sandwich ELISA. After treatment with or without tunicamycin (TM), culture supernatants were collected and analyzed. Streptavidin-phycoerythrin (SA-PE) was added for fluorescence detection, and the signal was quantified via flow cytometry. To perform the assay, capture beads were mixed with samples or standards, followed by incubation with detection reagents and PE-labeled antibodies. After washing, samples were analyzed using flow cytometry. The concentration of each cytokine was calculated using FCAP Array V3 software (BD Biosciences) based on standard curves.

### Macrophage differentiation and stimulation with conditioned medium

To prepare conditioned medium, WT-A549 and *PRKCSH*-KO A549 cells were cultured in the presence or absence of tunicamycin (TM, 1 µg/mL) for 48 h. The culture supernatants were then collected and centrifuged to remove debris, yielding conditioned media for downstream experiments.

For macrophage differentiation, THP-1 monocytes (ATCC) were seeded into 6-well plates at a density of 1 × 10⁶ cells per well and treated with 100 ng/mL phorbol 12-myristate 13-acetate (PMA; MCE) for 48 h to induce differentiation into M0 macrophages. After differentiation, M0 macrophages were cultured in the prepared conditioned media for an additional 48 h.

Afterward, cells were harvested for further analysis. Flow cytometry (NOVO CYTE 2070R, Agilent was performed to assess the proportion of M1 macrophages (CD45⁺ CD11b⁺ CD68⁺ CD86⁺) and M2 macrophages (CD45⁺ CD11b⁺ CD68⁺ CD163⁺). Additionally, real-time quantitative PCR (RT-qPCR) was conducted to evaluate the expression of M1- and M2-associated genes, including IRF5, STAT1, CEBPB, STAT6, IL-6, TNF-α, VEGF, and TGF-β.

### Macrophage subtype analysis in pleural effusion samples (Clinical Specimens)

Pleural effusion samples were collected from six patients with benign pleural effusion (BPE) and six with malignant pleural effusion (MPE). Each sample was centrifuged at 1,500 rpm for 10 min to isolate the cell pellet. The sedimented cells were stained for flow cytometry to evaluate macrophage subtypes.

Viable cells were first gated using live/dead discrimination dyes. Leukocytes were identified using CD45, and macrophages were gated using CD68. M1 macrophages were defined as CD45⁺CD68⁺CD86⁺, and M2 macrophages as CD45⁺CD68⁺CD163⁺. The percentage of each subtype was calculated relative to the parent CD45⁺CD68⁺ macrophage population. CD45 was incorporated into the staining panel to accurately identify leukocytes and differentiate them from tumor cells.

### Zebrafish xenograft model for tumor progression and immune modulation

The zebrafish xenograft model provides an efficient platform for studying tumor progression and innate immune responses due to its transparency, rapid development, and lack of adaptive immunity before 4–6 weeks post-fertilization [24]. This model allows for real-time visualization of tumor growth dynamics and macrophage-tumor interactions in response to PRKCSH deficiency. Zebrafish were maintained and bred at the Public Platform of Zebrafish Technology, Southwest Medical University, under standard husbandry conditions, following the Guide for the Care and Use of Laboratory Animals (NIH Publication No. 85 − 23). Adult zebrafish were housed at 28 °C under a 14/10-hour light/dark cycle and were fed freshly hatched fairy shrimp twice daily. Embryos were incubated at 28 °C in E3 medium supplemented with PTU (Macklin, China) to enhance transparency.

To assess tumor progression, WT-A549 and *PRKCSH*-KO A549 cells were stained with DiO fluorescent dye (Beyotime, China). At 48 h post-fertilization (hpf), dechorionated embryos were microinjected with stained tumor cell suspensions into the yolk sac using a precision microinjector. Embryos exhibiting sufficient fluorescent tumor signals (*n* ≥ 6/group) were selected and cultured. Tumor proliferation was monitored over three consecutive days using a confocal microscope (Olympus FV4000), and high-resolution images were captured to assess tumor dynamics.

To investigate macrophage recruitment and polarization in response to PRKCSH modulation, transgenic zebrafish [Tg(mpeg1:DsRed)] were used, which express red fluorescent protein (DsRed) under the *mpeg1* promoter, allowing for specific visualization of macrophages. WT-A549 and *PRKCSH*-KO A549 cells were pre-stained with DiO (a green-fluorescent membrane dye) and mixed with human PBMCs at a 1:9 ratio. At 48 hpf, embryos were dechorionated, and the tumor cell-PBMC mixtures were microinjected into the yolk sac. Embryos with sufficient fluorescence (*n* ≥ 6/group) were cultured for 48–72 h post-injection (hpi). Macrophage recruitment to tumor cells was monitored via confocal microscopy (Olympus FV4000), and high-resolution images were acquired to assess macrophage polarization.

To quantify macrophage polarization in zebrafish, we performed morphometric analysis of DsRed⁺ macrophages using high-resolution confocal imaging. Image analysis was conducted with ImageJ software to assess key shape parameters, including Feret diameter, aspect ratio, and circularity. Based on zebrafish cell size and shape, we used adjusted morphometric thresholds to sort individual macrophages into M1-like or M2-like groups. M1-like macrophages had a smaller Feret diameter (≤ 18 μm), a lower aspect ratio (≤ 2.0), and a higher circularity (≥ 0.65) consistent with rounder shape. On the other hand, M2-like macrophages had a Feret diameter of at least 23 μm, an aspect ratio of at least 2.5, and a circularity of at most 0.55, which is consistent with an elongated or bipolar shape. This method of classifying images was based on earlier research on mammalian macrophages that showed how useful quantitative shape descriptors, especially aspect ratio and circularity, can be for telling the difference between M1 and M2 phenotypes [25, 26]. Using these criteria, we quantified the proportion of M1 and M2 macrophages to assess the impact of PRKCSH deficiency on macrophage polarization in the zebrafish tumor microenvironment. Total RNA was also extracted from zebrafish embryos for downstream gene expression analysis, including real-time RT-PCR of human IFN-γ, Granzyme B, and PD-1 transcripts.

### Statistical analysis

Group comparisons were performed using independent t-tests for normally distributed data and Mann–Whitney U tests for nonparametric two-group comparisons. For multiple group comparisons, one-way ANOVA followed by Tukey’s post hoc test (for normally distributed data) or Kruskal–Wallis test with Dunn’s post hoc test (for nonparametric data) was applied as appropriate.

## Results

### Differential expression and dysregulated ER stress response in lung cancer tissues

**Fig. 1:**
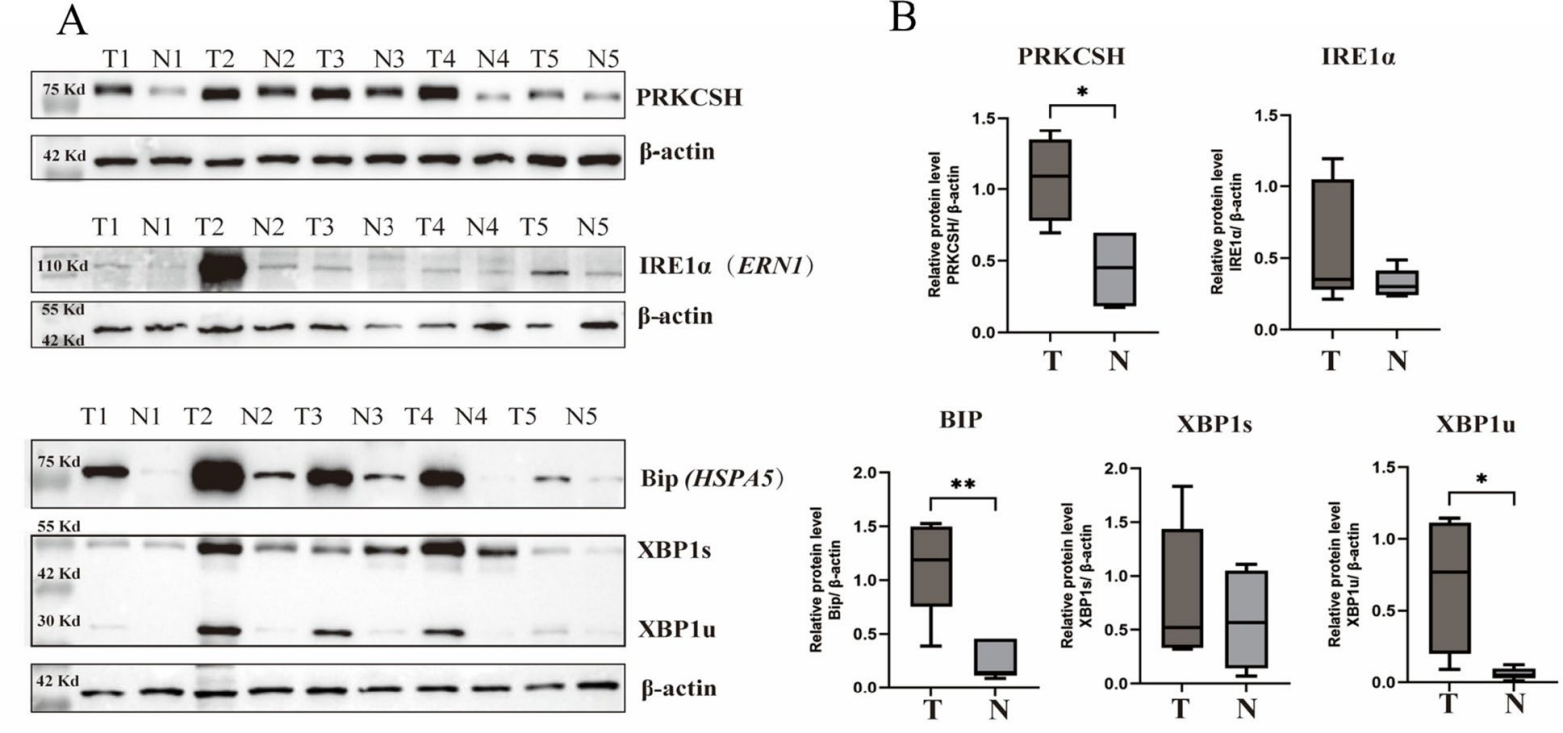
Expression of PRKCSH and ER Stress-Related Markers in Lung Adenocarcinoma Tissues. (**A**) Representative Western blot analysis of PRKCSH, IRE1α (ERN1), Bip (HSPA5), XBP1s, and XBP1u protein levels in tumor (T) and adjacent normal (N) tissues from lung adenocarcinoma patients. β-actin was used as a loading control. (**B**) Quantitative analysis of relative protein levels normalized to β-actin, presented as box plots depicting the median and interquartile ranges (*p < 0.05, **p < 0.01, Mann Whitney U test)

Protein expression levels of PRKCSH, IRE1α (ERN1), HSPA5 (BIP), and XBP1s were assessed in lung tumor tissues and nearby normal tissues using Western blot analysis. The expression of PRKCSH, IRE1α, and BIP is significantly higher in tumor (T) tissues than in normal (N) adjacent tissues (Fig. 1A). These results are further supported by quantitative analysis in Fig. 1B, which shows that tumor tissues had significantly higher expression levels (*p* < 0.01) of PRKCSH and HSPA5 (BIP) than nearby normal tissues. Similarly, although the difference was not as great, tumor tissues had higher levels of IRE1α (ERN1) expression. Tumor tissues also showed a significant upregulation of XBP1s, the spliced form of XBP1, indicating an activated unfolded protein response (UPR) within the tumor microenvironment. These results suggest a role for the ER stress signaling pathway in tumor-associated UPR activation by showing dysregulation and upregulation of ER stress signaling pathway components in lung tumor tissues, specifically PRKCSH and the IRE1α-XBP1 axis.

### PRKCSH overexpression and functional pathway analysis in lung adenocarcinoma

**Fig. 2:**
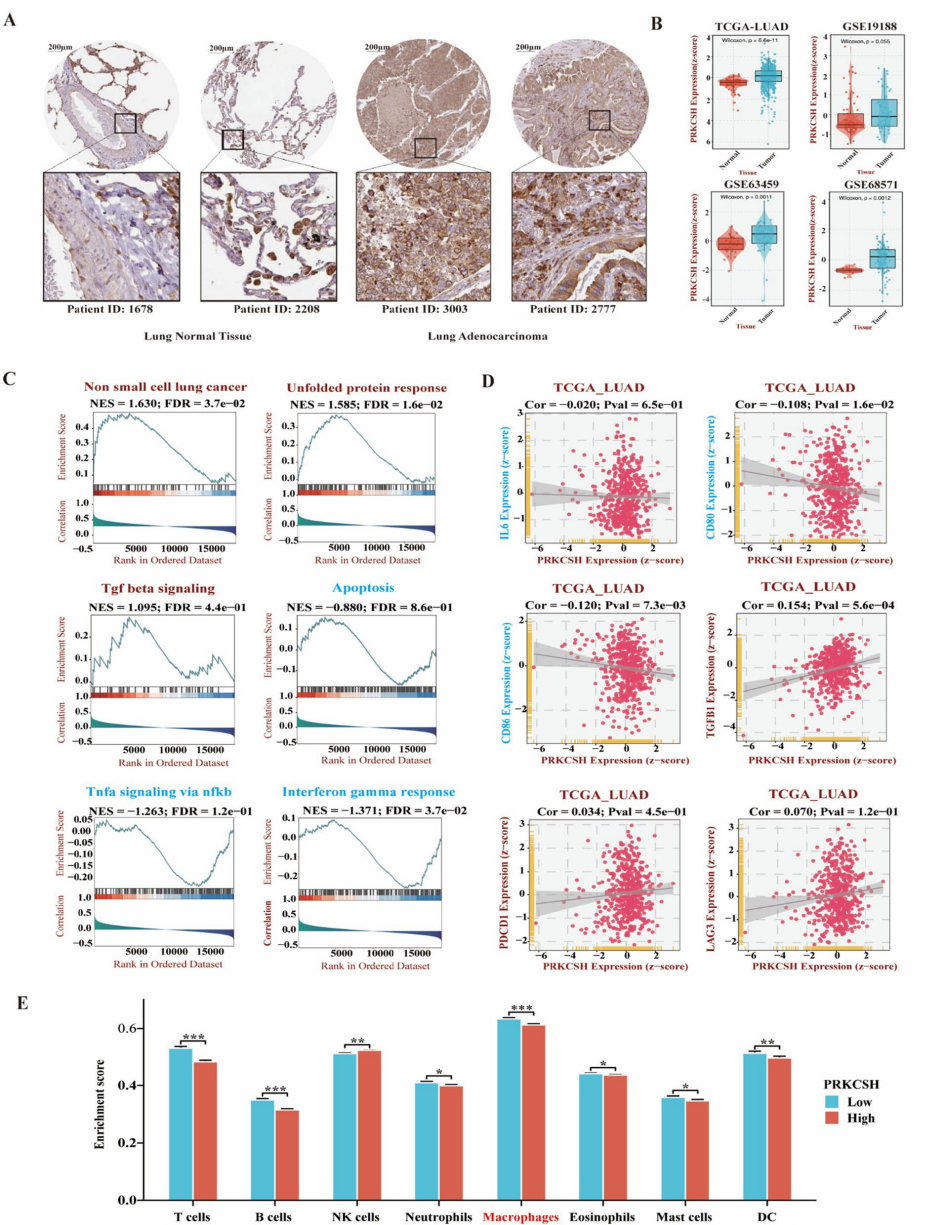
PRKCSH expression and its association with immune regulation and stress response in lung adenocarcinoma. **(A)** Representative immunohistochemistry (IHC) images from the Human Protein Atlas (HPA) showing PRKCSH protein expression in normal lung tissues (Patients 1678 and 2208) and lung adenocarcinoma tissues (Patients 3003 and 2777). Tumor tissues display markedly higher PRKCSH expression compared to normal tissues. **(B)** Box and violin plots displaying PRKCSH mRNA expression in tumor versus normal tissues across multiple datasets (GSE11117, GSE11969, GSE19188, GSE63459, GSE68571, and TCGA-LUAD). PRKCSH is significantly upregulated in tumors in most datasets, as assessed by Wilcoxon tests. **(C)** Hallmark Gene Set Enrichment Analysis (GSEA) comparing PRKCSH-high versus PRKCSH-low LUAD tumors. PRKCSH-high tumors are positively enriched in pathways including non-small cell lung cancer, unfolded protein response (UPR), TGF-β signaling, Negative enrichment is observed for apoptosis, TNFα signaling and interferon gamma response. **(D)** Correlation analysis between PRKCSH expression and immune-related genes in the TCGA-LUAD dataset. PRKCSH expression negatively correlates with CD80 and CD86—genes typically associated with immune activation. It is significantly positively correlated with TGFB1 and shows weak or non-significant associations with IL6, PDCD1 (PD-1), and LAG3 **(E)** Immune cell infiltration analysis comparing PRKCSH-high and PRKCSH-low LUAD tumors. High PRKCSH expression is associated with reduced infiltration of various immune cell types, including T cells, B cells, NK cells, neutrophils, eosinophils, and mast cells. Macrophages remain the most abundant immune population but are significantly lower in PRKCSH-high tumors

To validate the overexpression of PRKCSH in lung adenocarcinoma (LUAD), immunohistochemical (IHC) analysis using data from the Human Protein Atlas (HPA) and clinical tissue samples confirmed significantly higher PRKCSH expression in LUAD tissues compared to normal lung tissues (Fig. 2A). Gene expression analysis from GEO and TCGA datasets further supported this finding, with the most significant difference observed in the TCGA dataset (*p* = 6.6e-11) (Fig. 2B), positioning PRKCSH as a potential LUAD biomarker.

Hallmark Gene Set Enrichment Analysis (HGSEA) revealed that tumors with high PRKCSH expression are positively enriched for pathways related to non-small cell lung cancer, unfolded protein response, and Transforming Growth Factor Beta (TGF-β) signaling, suggesting a role for PRKCSH in tumor progression and immune regulation. In contrast, pathways associated with apoptosis, Tumor Necrosis Factor Alpha (TNF-α) signaling, and the interferon-gamma (IFN-γ) response were negatively enriched, indicating suppressed immune activation. Notably, TGF-β signaling is associated with M2 macrophage polarization, whereas TNF-α and IFN-γ are linked to M1 macrophage activity (Fig. 2C). Correlation analysis in the TCGA-LUAD cohort showed that PRKCSH expression was negatively correlated with CD86 and CD80, markers typically associated with M1 macrophages. By contrast, PRKCSH was significantly positively correlated with TGFB1, a cytokine linked to M2-like, immunosuppressive programming in the tumor microenvironment. IL6, programmed cell death protein 1 (PDCD1/PD-1), and lymphocyte-activation gene 3 (LAG-3) exhibited weak or non-significant associations with PRKCSH (Fig. 2D).

Immune cell profiling revealed that multiple immune cell types—including T cells, B cells, eosinophils, mast cells, and neutrophils—were significantly reduced in PRKCSH-high tumors. Despite this overall reduction, macrophages remained the most abundant immune cell type, suggesting that macrophages may play a dominant role in shaping the tumor immune landscape in PRKCSH-high LUAD cases (Fig. 2E). Based on these findings, we aim to further investigate how elevated PRKCSH expression influences macrophage polarization and function, particularly in the context of immune suppression and tumor progression.

### PRKCSH deficiency impairs cell proliferation, and migration

**Fig. 3:**
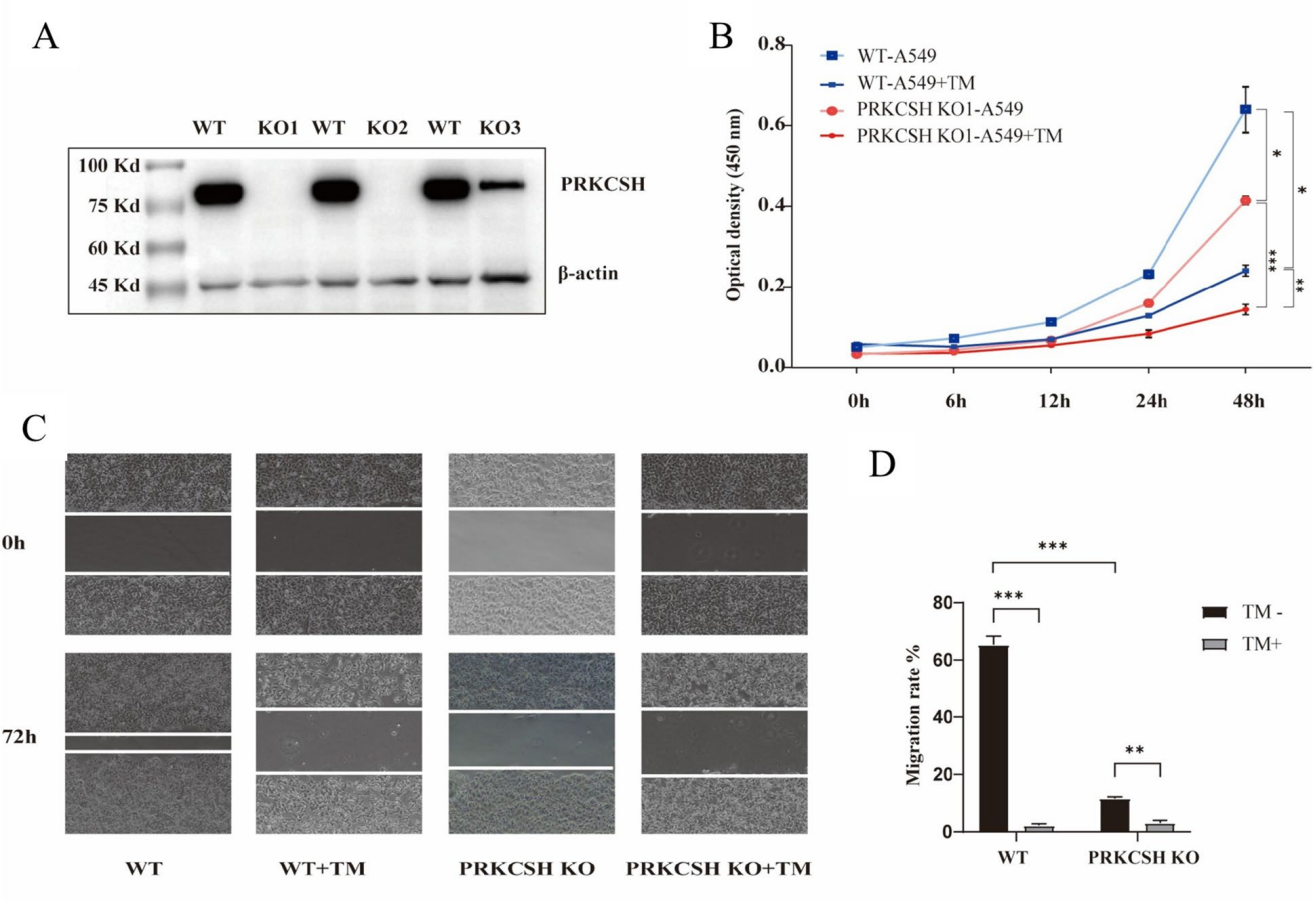
Impact of PRKCSH deficiency on A549 lung adenocarcinoma cell proliferation and migration. (**A**) Western blot confirming the successful knockout of *PRKCSH* in A549 cells (KO1 and KO2 show loss of *PRKCSH*; KO3 shows reduced PRKCSH vs. WT (β-actin control)). (**B**) Growth curves of WT A549 and *PRKCSH*-KO cells with/without tunicamycin (TM) treatment over 48 h, measured by CCK8-based method. (**C**) Wound healing assay images comparing migration of WT and *PRKCSH*-KO cells at 0 and 72 h, with and without TM treatment. (**D**) Bar graph quantifying migration ratio (72 h/0 h), showing significantly reduced migration in *PRKCSH*-KO cells, especially under TM treatment

*PRKCSH*-KO A549 cells showed significantly reduced PRKCSH protein levels, confirming successful knockout (Fig. 3A). Sanger sequencing confirmed frameshift mutations in KO1 (− 1 bp) and KO2 (+ 2 bp), whereas KO3 contained mixed alleles (see Supplementary Data S1, ZIP). KO1 and KO2 were therefore used for functional assays. Proliferation assays showed that *PRKCSH*-KO cells had reduced growth compared to WT A549 cells (*p* < 0.05), with a more pronounced decrease (*p* < 0.005) under TM treatment (Fig. 3B). Migration assays demonstrated that WT cells migrated more effectively than *PRKCSH*-KO cells. TM treatment significantly reduced migration in WT cells, while *PRKCSH*-KO cells exhibited minimal migration in both untreated and TM-treated conditions (Fig. 3C and D). These findings indicate that PRKCSH plays a critical role in promoting both cell proliferation and migration, especially under stress conditions.

### Enhanced susceptibility to apoptosis and ferroptosis but attenuated autophagy in *PRKCSH*-KO A549 cells under ER stress

**Fig. 4:**
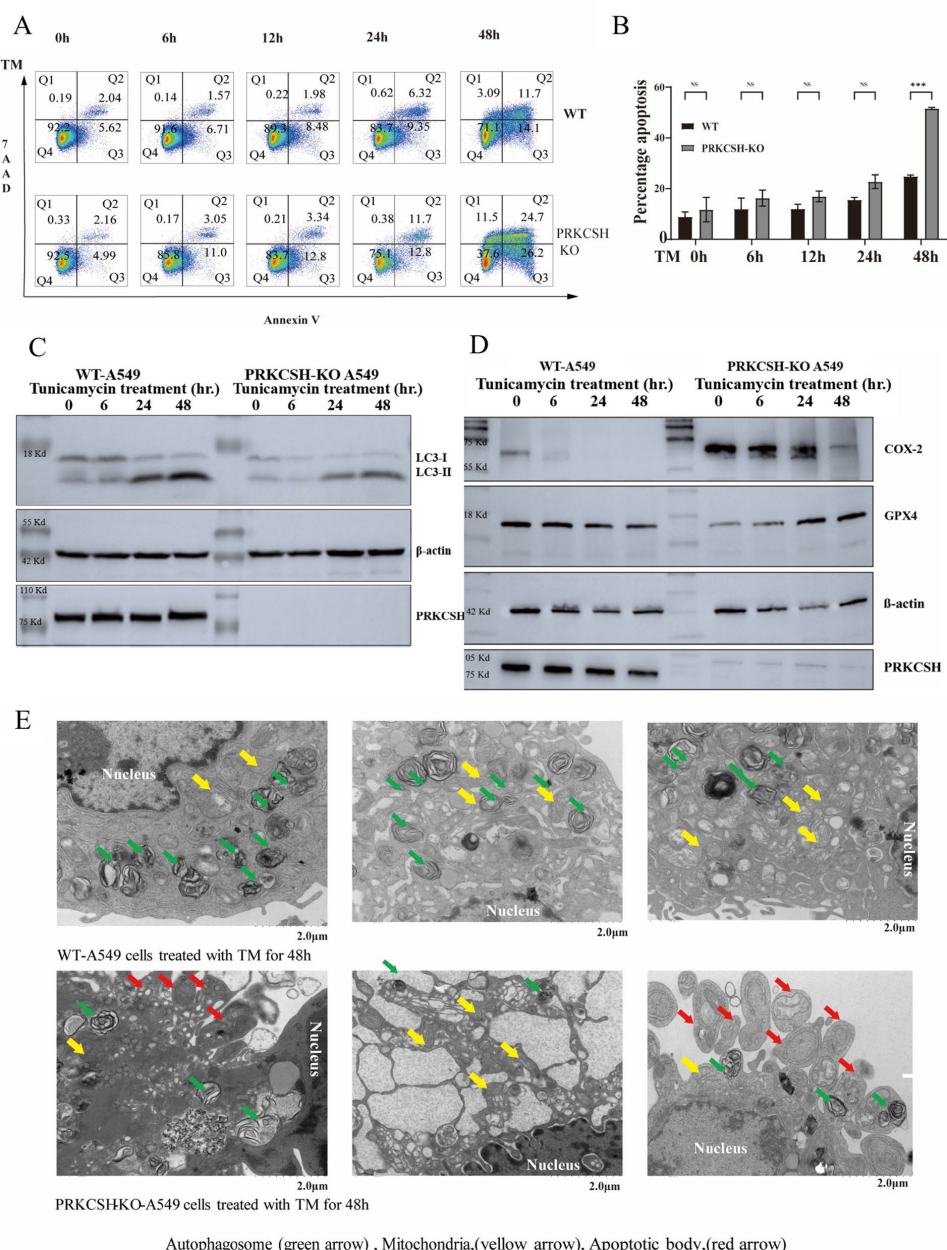
Impact of PRKCSH deficiency on apoptotic markers and ferroptosis in A549 lung adenocarcinoma cells treated with tunicamycin (TM). (**A**,** B**) Annexin V/7-AAD flow cytometry and bar graph indicating higher apoptosis in PRKCSH KO cells at 48 h (**C**,** D**) Western blot analysis of autophagy (LC3-I/LC3-II) and ferroptosis markers (COX-2, GPX4) in WT and *PRKCSH*-KO A549 cells treated with tunicamycin. *PRKCSH*-KO cells show reduced autophagic flux (lower LC3-II levels) and increased ferroptosis susceptibility (elevated COX-2 and decreased GPX4 levels), indicating impaired adaptive responses to ER stress. (**E**) Transmission electron microscopy images of WT-A549 and *PRKCSH*-KO A549 cells treated with tunicamycin for 48 h. WT cells show abundant autophagosomes (green arrows) and intact mitochondria (yellow arrows), indicating active autophagy. *PRKCSH*-KO cells exhibit fewer autophagosomes and increased apoptotic bodies (red arrows), highlighting impaired autophagic response and heightened apoptosis under ER stress

Flow cytometry analysis (Fig. 4A and B) revealed a significant increase in Annexin V-positive apoptotic cells in *PRKCSH*-KO A549 cells, especially at 48 h post-treatment (*p* < 0.001). Markers of ferroptosis further indicated heightened susceptibility in *PRKCSH*-KO cells. Figure 4D demonstrates increased baseline levels of COX-2 and a sharper decline in GPX4 levels in *PRKCSH*-KO cells following TM treatment compared to WT-A549 cells, signifying elevated ferroptotic activity under ER stress conditions. Interestingly, autophagic responses were notably attenuated in *PRKCSH*-KO cells compared to WT controls. Although LC3-I to LC3-II conversion, a hallmark of autophagy, was observed in both cell types, *PRKCSH*-KO cells exhibited significantly reduced LC3-II levels under TM-induced stress (Fig. 4C). This suggests a compromised autophagic response in *PRKCSH*-KO cells during ER stress.

Ultrastructural analysis via electron microscopy (Fig. 4E) was consistent with these findings, revealing fewer autophagosomes (green arrows) in *PRKCSH*-KO cells compared to WT cells. In contrast, mitochondrial abnormalities (yellow arrows) and apoptotic bodies (red arrows) were more prominent in *PRKCSH*-KO cells, reflecting increased apoptotic activity despite diminished autophagic engagement. Together, these results indicate that *PRKCSH*-KO cells exhibit heightened susceptibility to apoptosis and ferroptosis, coupled with an impaired autophagic response under ER stress.

### Impact of PRKCSH deficiency on UPR and stress response pathways in A549 cells

**Fig. 5:**
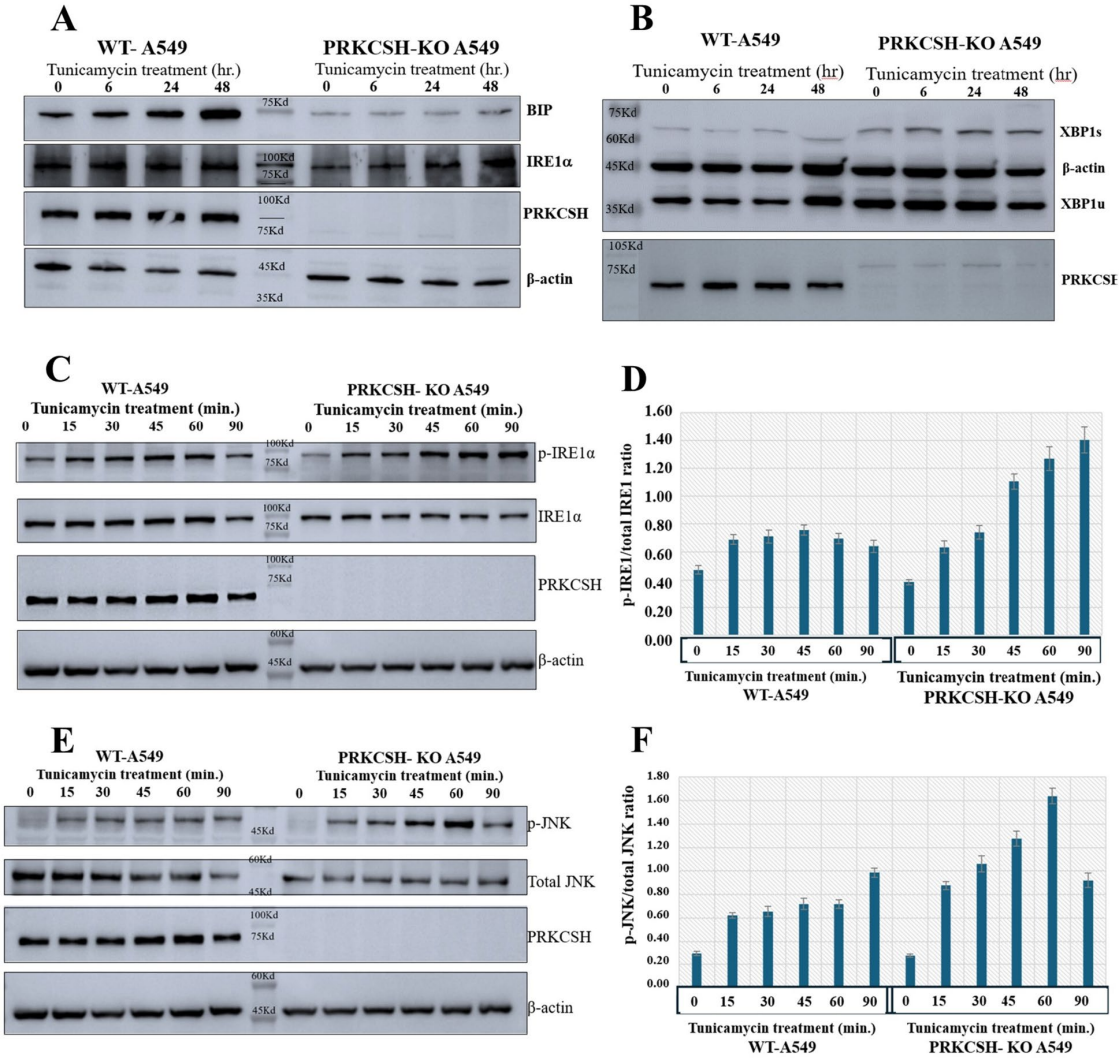
Impact of PRKCSH deficiency on IRE1α signaling pathway in A549 cells under ER stress. (**A**) Western blot analysis showing the expression levels of BIP, IRE1α, PRKCSH, and β-actin in WT and *PRKCSH*-KO A549 cells treated with tunicamycin over 0, 6, 24, and 48 h. (**B**) Western blot analysis showing XBP1 splicing (XBP1s and XBP1u) in WT and *PRKCSH*-KO A549 cells after tunicamycin treatment over 0, 6, 24, and 48 h. (**C**) Western blot analysis of phosphorylated IRE1α (p-IRE1α) and total IRE1α in WT and *PRKCSH*-KO A549 cells over 90 min of tunicamycin treatment. (**D**) Quantification of p-IRE1α/total IRE1α ratio from (**C**), showing a time-dependent increase in phosphorylated IRE1α in *PRKCSH*-KO cells. (**E**) Western blot analysis of phosphorylated JNK (p-JNK) and total JNK in WT and *PRKCSH*-KO A549 cells over 90 min of tunicamycin treatment. (**F**) Quantification of p-JNK/total JNK ratio from (**E**), indicating heightened JNK activation in *PRKCSH*-KO cells under ER stress conditions.

In contrast to WT-A549 cells, where BIP expression dramatically increased over time, A549 cells with PRKCSH deficiency had an impaired ER stress response, as evidenced by decreased basal expression of BIP and its inability to increase upon tunicamycin treatment (Fig. 5A). On the other hand, *PRKCSH*-KO cells had lower levels of IRE1α expression when they were at rest, but this was greatly increased after receiving tunicamycin, especially at 24 and 48 h, suggesting a compensatory response. When comparing *PRKCSH*-KO cells to WT, increased XBP1 splicing (XBP1s) was seen, indicating increased IRE1α activation (Fig. 5B).

*PRKCSH*-KO cells had lower basal phosphorylation levels of both proteins than WT cells, according to additional analysis of IRE1α (ERN1) phosphorylation and its downstream effector JNK (JKAMP) during a 90-minute tunicamycin treatment. Yet, IRE1α and JNK phosphorylation dramatically rose in *PRKCSH*-KO cells following tunicamycin treatment, suggesting a robust but delayed activation of the UPR and stress response (Fig. 5C-F).

These findings indicate a crucial role for PRKCSH in maintaining a balance between ER stress responses, as *PRKCSH*-KO causes a compensatory overactivation of the IRE1 pathway in addition to impairing the ER stress response through decreased BIP expression. Potential treatment options that target IRE1 signaling in cancer cells lacking PRKCSH are highlighted by this dysregulation.

### PRKCSH deficiency suppresses IL-6 and IL-8 under ER stress, and single-cell transcriptomic analysis links PRKCSH to an M2-Like macrophage phenotype

**Fig. 6:**
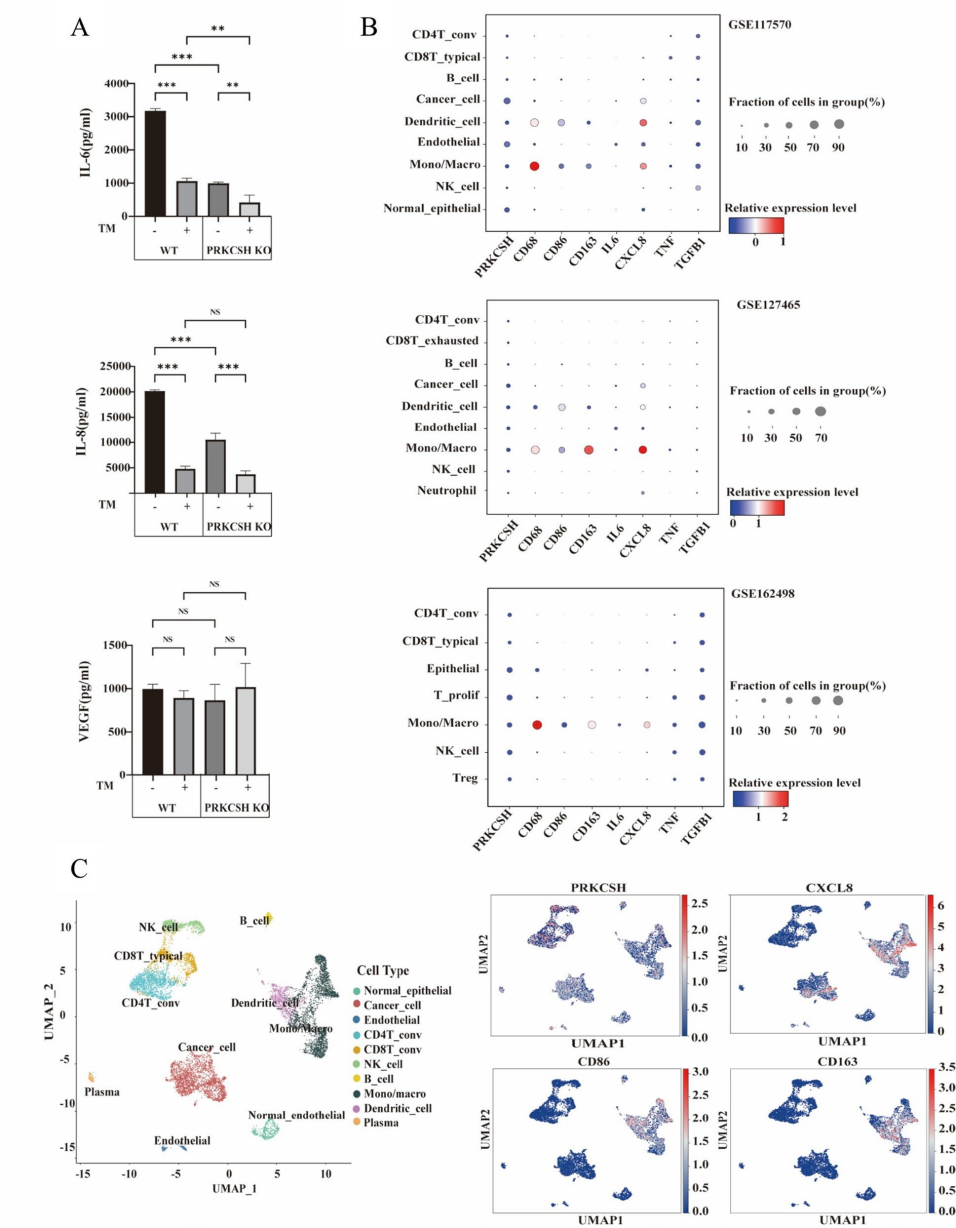
PRKCSH deficiency alters cytokine secretion and immune landscape in lung adenocarcinoma. (**A**) CBA of cytokines secreted by WT and *PRKCSH*-KO A549 lung adenocarcinoma cells with or without tunicamycin (TM) treatment. Conditioned media were analyzed for IL-6, IL-8, and vascular endothelial growth factor (VEGF) concentrations. Data represent mean ± SD from three independent experiments. Statistical comparisons were performed using one-way ANOVA with Tukey’s multiple comparisons test. **p < 0.01; *p < 0.005; ns, not significant. (**B**) Dot plot visualization of cell-type-specific gene expression across three single-cell RNA sequencing (scRNA-seq) datasets (GSE127465, GSE117570, GSE162498). Cell types are shown on the y-axis and genes on the x-axis. Dot size reflects the percentage of cells expressing the gene, and color intensity indicates average gene expression level. PRKCSH expression and its association with immune markers (CD80, CD86, CD163), cytokines (IL6, IL8, CCL4, TNF), and immunosuppressive factor TGFB1 are highlighted, with notable enrichment in monocyte/macrophage (Mono/Macro) clusters. (**C**) Uniform Manifold Approximation and Projection (UMAP) plots of integrated scRNA-seq GSE117570 datasets showing the spatial distribution of major cell populations (left) and expression of selected genes (right). Feature plots demonstrate the expression of PRKCSH, CXCL8 (IL8), CD86, and CD163, with prominent expression in monocyte/macrophage populations, reinforcing their potential role in modulating the tumor immune microenvironment

We measured the secretion levels of VEGF, IL-6, and IL-8 in wild-type and *PRKCSH*-KO A549 cells treated with or without tunicamycin (TM) in order to investigate the function of PRKCSH in cytokine regulation during endoplasmic reticulum (ER) stress. TM treatment and PRKCSH deficiency both significantly decreased IL-6 and IL-8 secretion, as seen in Fig. 6A. These cytokines were produced in high concentrations by WT cells at rest, but after TM treatment, they were significantly reduced (*p* < 0.001). Compared to WT, *PRKCSH*-KO cells had lower baseline secretion, and TM further reduced their levels of IL-6 and IL-8 (*p* < 0.01 and *p* < 0.001, respectively). These results suggest that ER stress and PRKCSH deficiency work together to reduce the production of pro-inflammatory cytokines. On the other hand, VEGF secretion did not change in any of the groups, indicating that the effects were specific.

Using the scCancerExplorer platform, we examined three single-cell RNA sequencing datasets (GSE127465, GSE117570, and GSE162498) to investigate the relationship between PRKCSH expression and macrophage phenotypes within the tumor microenvironment. Dot plots of the Mono/Macro cell cluster across all datasets demonstrated high expression of IL8 and CD163, with M2 macrophage marker CD163 displaying especially consistent expression (Fig. 6B). These results imply that M2-like macrophages are more prevalent in the tumour microenvironment and attribute their polarization to IL8.

On the GSE117570 dataset, dimensionality reduction using Uniform Manifold Approximation and Projection (UMAP) (Fig. 6C) showed discrete cellular clusters with co-localized expression of IL8 and CD163 within the Mono/Macro population. A correlation between high PRKCSH expression and a tendency towards an immunosuppressive, M2-like macrophage phenotype was further supported by the finding that CD86, a marker of M1 macrophages, displayed little spatial overlap with IL8.

### PRKCSH deficiency functionally alters the tumor secretome to promote M1 macrophage polarization

Based on what we learned from before about how PRKCSH expression is linked to an M2-like macrophage phenotype, we wanted to evaluate how PRKCSH deficiency affected the polarization of macrophages. We did macrophage stimulation tests with conditioned media from A549 tumor cells (WT and *PRKCSH*-KO) to investiagte how the macrophage phenotype changed. Then we used RT-qPCR and flow cytometry to evaluate how the phenotype changed. Macrophages from THP-1 cells that were treated with conditioned media from *PRKCSH*-KO A549 cells showed big changes in phenotype compared to those that were treated with WT-derived media.

**Fig. 7:**
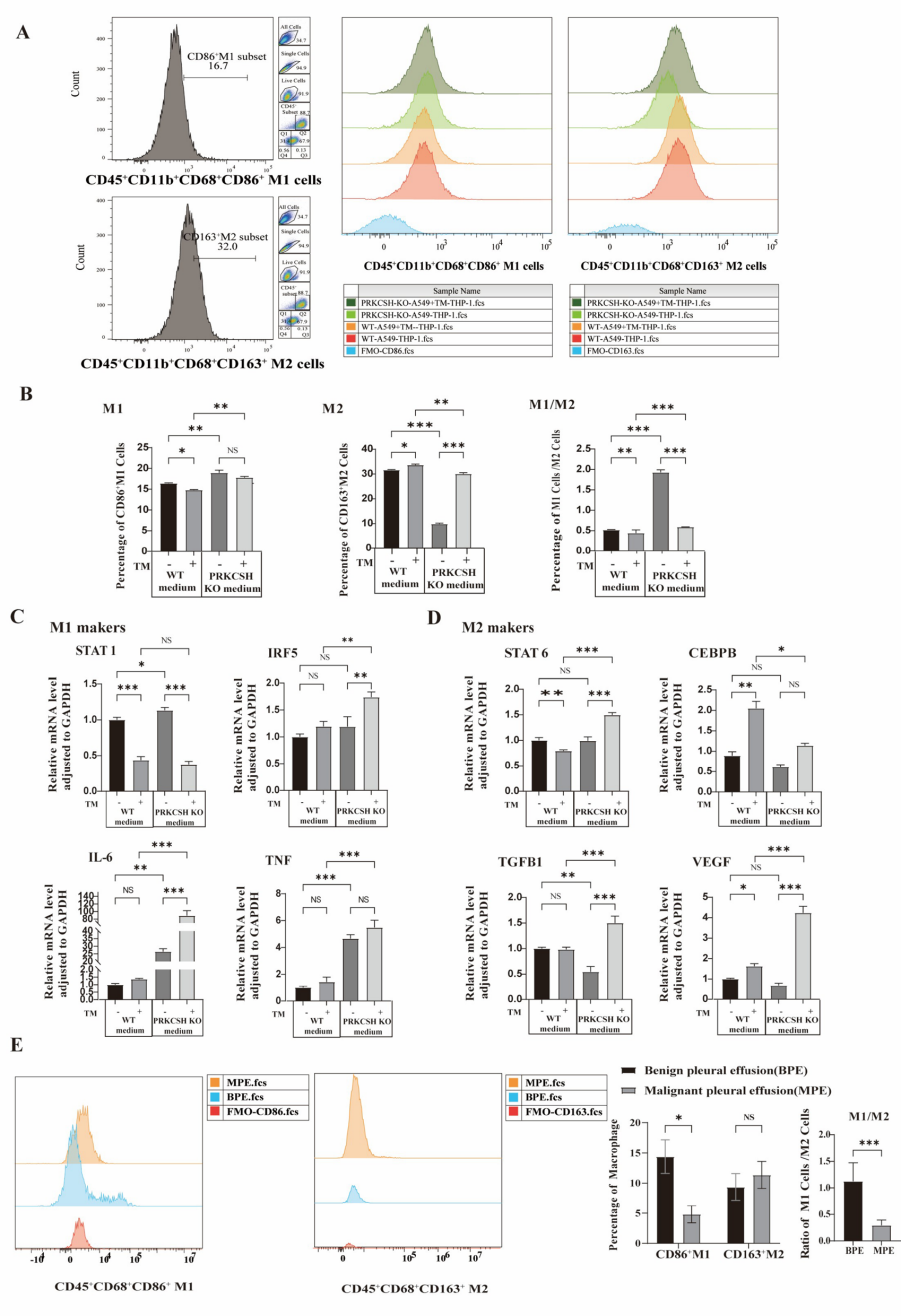
Effect of tumor-derived conditioned medium from *PRKCSH*-KO cells on macrophage polarization. **(A)** Flow cytometry histograms showing CD86 (M1 marker) and CD163 (M2 marker) expression in THP-1-derived macrophages stimulated with conditioned medium from WT or *PRKCSH*-KO A549 cells under tunicamycin (TM) treatment. Conditioned medium from *PRKCSH*-KO cells increased the proportion of CD86⁺ M1 macrophages and decreased CD163⁺ M2 macrophages. **(B)** Quantification of flow cytometry data showing a significant increase in CD86⁺ M1 macrophages and a corresponding decrease in CD163⁺ M2 macrophages in the *PRKCSH*-KO group. The M1/M2 ratio was significantly elevated in macrophages treated with *PRKCSH*-KO conditioned medium, indicating a shift toward a pro-inflammatory phenotype. Data are presented as mean ± SD; ns = not significant, *p < 0.05, **p < 0.01, ***p < 0.005 (Mann–Whitney U test). **(C)** RT-qPCR analysis of M1-associated genes (IRF5, STAT1, TNF-α, IL-6) in macrophages stimulated with conditioned medium from WT or *PRKCSH*-KO A549 cells with or without tunicamycin (TM). *PRKCSH*-KO conditioned medium significantly upregulated IRF5, TNF-α, and IL-6, while STAT1 expression was reduced under TM treatment. **(D)** RT-qPCR analysis of M2-associated genes (STAT6, CEBPB, TGF-β, VEGF) in macrophages treated with conditioned medium from WT or *PRKCSH*-KO cells, with or without tunicamycin (TM). Without TM, TGF-β was significantly lower with *PRKCSH*-KO conditioned medium, while STAT6, CEBPB, and VEGF showed no difference. With TM, STAT6, TGF-β, and VEGF were higher and CEBPB was lower with PRKCSH-KO conditioned medium compared with WT. **(E)** Flow cytometric analysis of pleural effusion samples from patients with benign (BPE) and malignant (MPE) conditions. CD86⁺ M1 macrophages were significantly enriched in BPE samples, whereas the proportion of CD163⁺ M2 macrophages did not differ significantly. The M1/M2 ratio was significantly reduced in MPE samples, supporting a shift toward an M2-dominant phenotype in the tumor microenvironment

Figure 7A and B show that flow cytometry analysis showed big changes in THP-1-derived macrophages subpopulations when *PRKCSH* was knockout. When PRKCSH was knocked out, there were more M1 macrophages (CD45⁺CD11b⁺CD68⁺CD86⁺) and fewer M2 macrophages (CD45⁺CD11b⁺CD68⁺CD163⁺). Quantitative analysis (Fig. 7B) showed that there were a lot more CD86⁺ M1 macrophages in *PRKCSH*-KO conditions (*p* < 0.01) and a lot fewer CD163⁺ M2 macrophages (*p* < 0.005). When *PRKCSH* was knockout, the M1/M2 ratio shifted strongly toward an M1-dominant phenotype (*p* < 0.005), which supports a shift toward a pro-inflammatory state.

Gene expression analysis (Fig. 7C and D) backed up this change in polarization even more. When macrophages were resting, those that were exposed to *PRKCSH*-KO-conditioned medium had much higher levels of STAT1 (*p* < 0.05), IL-6 (*p* < 0.01), and TNF-α (*p* < 0.005) than those that were treated with WT-conditioned medium. This suggests that the cells were moving toward an M1-like phenotype. The expression of IRF5 did not change (*p* > 0.05), which means that it needs more stimulation to be activated. When TM caused ER stress, macrophages treated with *PRKCSH*-KO-conditioned medium showed a big increase in IRF5 (*p* < 0.01) and even more IL-6 and TNF-α expression (*p* < 0.005), which strengthened M1 polarization. It’s interesting that STAT1 levels went down in both groups when they were treated with TM. This shows that TM can suppress STAT1 levels regardless of *PRKCSH* status.

When examining M2-associated markers, there were no major differences in the levels of STAT6 or CEBPB between the *PRKCSH*-KO and WT groups when they were at rest. However, when TM was added, STAT6 levels were significantly increased in the *PRKCSH*-KO group (*p* < 0.005), while CEBPB levels were decreased compared to the TM-treated WT controls (*p* < 0.05). TGF-β levels were much lower in macrophages that had been treated with *PRKCSH*-KO when they were not stressed (*p* < 0.01), but both TGF-β and VEGF levels went up a lot when they were stressed (*p* < 0.001). This shows that stress reactivated M2 cytokine pathways.

To confirm these results in a clinical setting, we examined the polarization profiles of macrophages in pleural effusion samples from patients with benign (BPE) and malignant (MPE) conditions. Flow cytometry showed that BPE samples had a lot more CD86⁺ M1 macrophages than MPE samples, but CD163⁺ M2 macrophages did not differ much. The M1/M2 ratio was much lower in MPE, which supports the idea that the malignant setting is shifting toward an M2-dominant, immunosuppressive phenotype (Fig. 7E). These clinical observations support what we found in vitro and show how important PRKCSH is in creating an immune microenvironment that allows tumors to grow.

### Zebrafish xenograft model validates *PRKCSH* deficiency effects on tumor progression and immune modulation

**Fig. 8:**
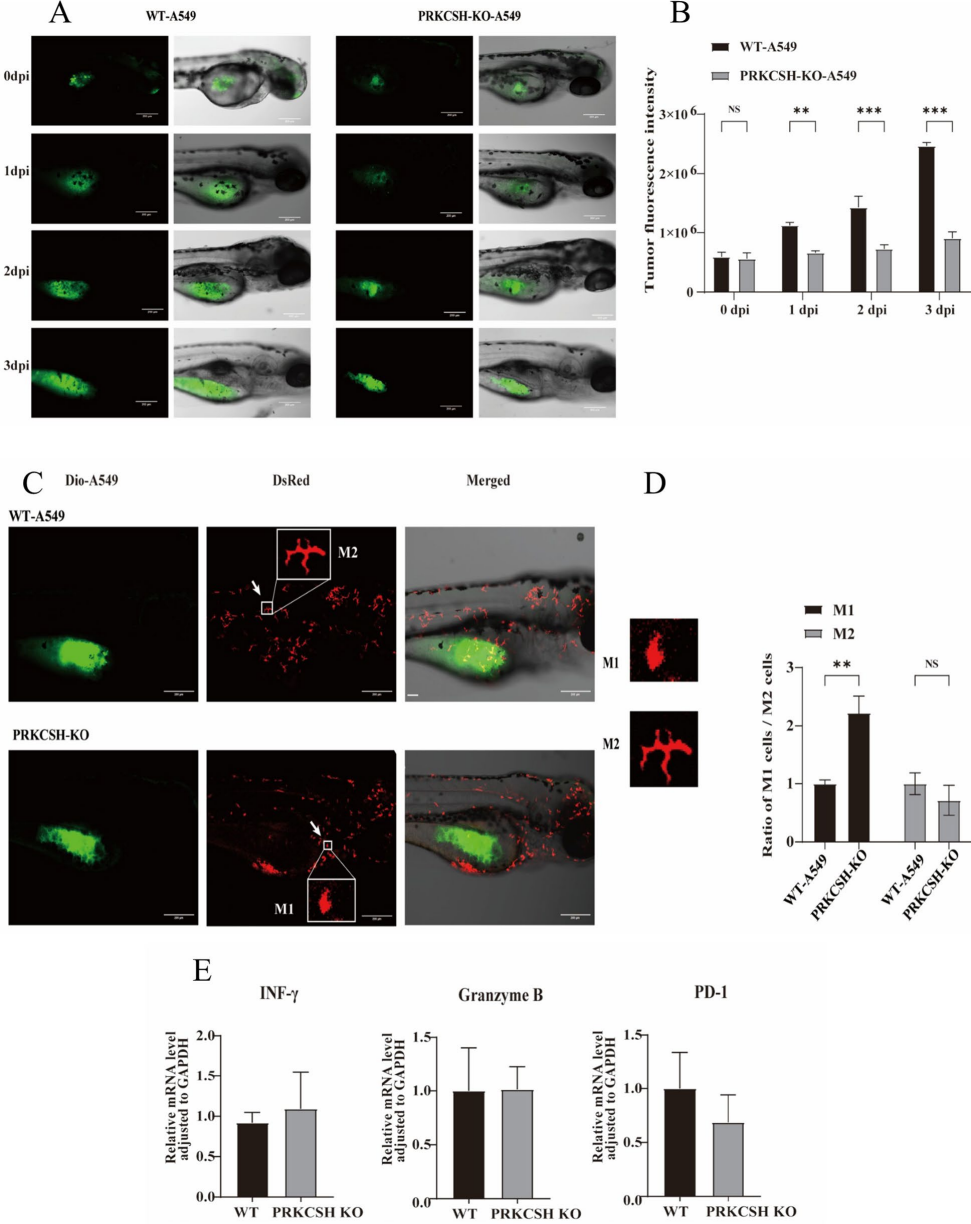
Assessment of tumor proliferation and macrophage polarization using a zebrafish xenograft model. (**A**) Representative confocal images of zebrafish xenografts injected with either WT-A549 or *PRKCSH*-KO A549 cells at 0-, 1-, 2-, and 3-days post-injection (dpi). Each group consisted of 6 zebrafish. Left panels show fluorescence images, and right panels show merged fluorescence and brightfield images. Scale bar = 100 μm. (**B**) Quantification of tumor fluorescence intensity over three days. *PRKCSH*-KO A549 cells showed significantly reduced tumor fluorescence and size compared to WT-A549, indicating that PRKCSH deficiency inhibits tumor growth. Data are presented as mean ± SD; ns = not significant, *p* < 0.05 (*), *p* < 0.01 (**), *p* < 0.005 (***) according to Mann Whitney U tests). (**C**) Confocal fluorescent imaging of macrophage polarization in zebrafish xenografts co-cultured with WT-A549 and *PRKCSH*-KO A549 cells. Green fluorescence (DiO) represents tumor cells, while red fluorescence (DsRed) highlights macrophages. In WT-A549 tumors, a higher proportion of anti-inflammatory M2 macrophages is observed, while *PRKCSH*-KO A549 tumors show increased pro-inflammatory M1 macrophages. The merged images indicate tumor-macrophage interactions. Polarization analysis confirms a higher M1/M2 ratio in *PRKCSH*-KO tumors, indicating a shift towards a pro-inflammatory phenotype. Scale bar = 20 μm. (**D**) Quantitative analysis of macrophage polarization based on morphometric profiling of DsRed⁺ macrophages in zebrafish xenografts. Using shape-based criteria (Feret diameter, aspect ratio, and circularity), macrophages were classified as M1-like or M2-like. PRKCSH deficiency significantly increased the proportion of M1-like macrophages compared to WT-A549, while M2-like macrophage levels remained unchanged. Data are shown as mean ± SD from at 6 embryos per group; *p* < 0.01, ns = not significant (Mann Whitney U test). (**E**) Relative mRNA expression levels of T cell activity markers in co-injected human PBMC (INF-γ, Granzyme B, and PD1) in WT-A549 and *PRKCSH*-KO A549 zebrafish xenografts. Although, no significant differences were observed in INF-γ or Granzyme B expression between groups, indicating similar T cell activation and cytotoxic potential, PD1 expression showed a downward trend in *PRKCSH*-KO A549 xenografts, suggesting reduced T cell exhaustion. Data are normalized to GAPDH and presented as mean ± SD

We used a zebrafish xenograft model that was grown with human PBMCs to verify our in vitro results and investigated what happens when *PRKCSH* is knockout in a living environment. This model let us see how tumors grow, how T cells respond in real time, and how macrophages are recruited and polarized. Fluorescent imaging (Fig. 8A) showed that *PRKCSH*-KO A549 tumors had much lower fluorescence intensity and smaller tumors. On the other hand, WT-A549 tumors grew strongly over three days after injection (dpi). Quantitative analysis (Fig. 8B) showed that there was no difference at 0 dpi. However, *PRKCSH*-KO tumors showed a gradual decrease in fluorescence starting at 1 dpi (*p* < 0.01), and the difference became more noticeable at 2 and 3 dpi (*p* < 0.001). These results back up the idea that *PRKCSH* could be a good target for lung cancer treatment because knocking it out stops tumors from growing in living things.

We injected DiO-labeled WT-A549 or *PRKCSH*-KO A549 cells into transgenic zebrafish embryos that had DsRed-labeled macrophages to investigate how *PRKCSH* expression affected the recruitment and polarization of macrophages in vivo. Figure 8C shows that confocal microscopy clearly showed how macrophages moved into the area 48 h after injection (hpi). The macrophages in *PRKCSH*-KO xenografts were round and compact, which is a sign of M1-like polarization. On the other hand, the macrophages in WT-A549 xenografts were long and branched, which is a sign of M2-polarized macrophages. We used morphometric changes, which were adopted from previous studies in mammalian model [25, 26] to measure the polarization of DsRed⁺ macrophages. The Feret diameters of M1-like macrophages were smaller (≤ 18 μm), the aspect ratios were lower (≤ 2.0), and the circularities were higher (≥ 0.65). The Feret diameters of M2-like macrophages were bigger (≥ 23 μm), their aspect ratios were higher (≥ 2.5), and their circularities were lower (≤ 0.55).

The percentage of M1-like macrophages was much higher in embryos transplanted with *PRKCSH*-KO A549 cells than in embryos that received WT-A549 cells (*p* < 0.01). Nevertheless, the two groups did not have a clear difference in the number of M2-like macrophages. These results suggest that tumor cells that PRKCSH deficiency selectively increase the recruitment or polarization of macrophages toward an M1-like phenotype without changing the number of M2-like macrophages significantly (Fig. 8D). This means that losing PRKCSH in vivo creates a tumor microenvironment that promotes immune response. We examined the levels of INF-γ, Granzyme B, and PD-1 in human PBMCs that had been injected with either WT-A549 or *PRKCSH*-KO A549 cells to investigate how T cells killed tumor cells and how the immune system was activated. There was no statistically significant difference in the levels of INF-γ, Granzyme B, and PD-1. However, *PRKCSH*-KO tumors tended to have lower PD-1 expression (Fig. 8E), which could mean that the immune system is less exhausted. These findings suggest that PRKCSH expression promotes M2 polarization, which makes the tumor microenvironment more immunosuppressive. However, when *PRKCSH* is knockout, it increases macrophage infiltration and shifts macrophage differentiation toward an M1-like phenotype, which boosts a pro-inflammatory anti-tumor immune response.

## Discussion

According to this study, PRKCSH plays a significant role in tumor progression and immune modulation regulation of lung adenocarcinoma. PRKCSH facilitates tumor cell survival and immune evasion by modulating cytokine secretion, macrophage polarization, and ER stress responses. We showed using zebrafish xenografts that PRKCSH knockout (KO) causes a reprogramming of the tumor immune microenvironment, which is characterized by an elevated M1/M2 macrophage ratio, and hinders tumor proliferation in vivo. The change to an M1 phenotype emphasizes how PRKCSH shapes macrophage function and raises the possibility that it could help reverse the M2-driven immune suppression that tumors frequently exhibit [27].

Mechanistically, PRKCSH appears to function as a homeostatic regulator of the IRE1α signaling axis. Under basal conditions, PRKCSH KO cells showed reduced phosphorylation of IRE1α and JNK, but upon ER stress induction, these responses were markedly amplified, including enhanced XBP1 splicing. This pattern of low basal but exaggerated stress-induced activation suggests that PRKCSH sets a threshold for IRE1α responsiveness, buffering cells against excessive ER stress signaling. Dysregulation of this axis may have downstream consequences for cytokine production, macrophage polarization, and stress-related cell fate decisions. The production of cytokines, macrophage polarization, and stress-related cell fate decisions may all be negatively impacted by dysregulation of this axis. The reduction of IL-6 and IL-8 expression in PRKCSH-KO cells is among the most noteworthy results, indicating that PRKCSH positively regulates these pro-inflammatory cytokines. In order to shape the tumor microenvironment (TME), both IL-6 and IL-8 are essential. Through STAT3 and STAT6 activation, IL-6 stimulates M2 macrophage polarization, tumor growth, and immune suppression [28, 29]. Additionally, it reduces antitumor immunity mediated by T cells [30]. IL-8 promotes angiogenesis, the epithelial–mesenchymal transition (EMT), metastasis, and the recruitment of neutrophils and macrophages. It is also linked to immunotherapy resistance [31]. By activating the MAPK and NF-κB pathways, which are downstream of neurotensin (NTS) signaling, IL-8 has been demonstrated to promote EMT and tumor invasion in hepatocellular carcinoma (HCC). A pro-oncogenic inflammatory microenvironment that promotes metastasis was promoted by HCC-derived IL-8, which also increased M2 polarization [32].

Our study demonstrated that PRKCSH plays a crucial role in maintaining cytokine-driven immunosuppressive signaling, which may otherwise promote tumor progression in part through M2 reprogramming. This is demonstrated by the suppression of IL-6 and IL-8 that was observed upon PRKCSH deficient or ER stress (TM treatment). Although the IRE1–XBP1 pathway has been reported to support M2 polarization and fibrotic remodeling in other contexts [33, 34], our data indicate that its hyperactivation in PRKCSH-KO cells under ER stress instead amplifies pro-inflammatory and apoptotic signaling through concurrent activation of the IRE1–JNK branch. By encouraging ER expansion in macrophages, IL-6 is known to support this process by boosting the secretory capacity and protein synthesis of these cells. Additionally, tumor cells can transmit ER stress to macrophages in the vicinity, which sets off inflammatory signals that support the preservation of a tumor-friendly environment [35].

By controlling IRE1α activity and IL-6/IL-8 secretion, these results imply that PRKCSH influences macrophage behavior and the inflammatory landscape of the tumor microenvironment in an indirect but significant way. This notion is supported by our co-culture experiments and gene-expression analyses, which revealed that macrophages shifted toward a pro-inflammatory, anti-tumor phenotype when PRKCSH was absent. Under tunicamycin-induced ER stress, STAT6, TGF-β, and VEGF transcripts increased, whereas CEBPB was reduced; this pattern is most consistent with a mixed stress-adaptive response rather than classical IL-4/IL-13-driven M2 polarization. Functional activation of M2 macrophages typically requires phospho-STAT6 (Tyr641) [36] and downstream target induction, which were not assessed in this study. In contrast, M1-associated markers (STAT1, IRF5, TNF-α, and IL-6) were significantly upregulated, reinforcing the notion that PRKCSH deficiency favors a pro-inflammatory, M1-skewed environment. Consistent with these findings, CD86⁺ M1 macrophage proportions were substantially higher in benign pleural effusion (BPE) samples than in malignant pleural effusion (MPE) samples, while CD163⁺ M2 macrophage levels were comparable between groups. The resulting decrease in the M1/M2 ratio in MPE underscores a shift toward an immunosuppressive tumor microenvironment and supports the translational relevance of PRKCSH-mediated immune modulation.

Along with immune modulation, loss of PRKCSH made tumor cells more vulnerable to ER stress-induced cell death, such as apoptosis and ferroptosis, while also compromising autophagy. This is the first study that we are aware of that connects PRKCSH to the control of ferroptosis. PRKCSH KO cells showed elevated COX-2 expression and decreased GPX4, which is consistent with increased ferroptotic susceptibility. Decreased autophagosome formation and LC3-II conversion also indicated a decrease in autophagic flux. Autophagy promotes immune evasion and tumor survival under stress [37, 38], so its disruption could further impair tumor adaptability.

These results are consistent with new research that links tumor immune evasion and PRKCSH overexpression. In lung cancer, PRKCSH controls cell adhesion molecules (CAMs), which are essential for T-cell surveillance [9],], stabilizes the insulin-like growth factor 1 receptor (IGF1R), and increases resistance to tumor necrosis factor superfamily (TNFSF)-induced apoptosis [15]. Furthermore, across a variety of malignancies, PRKCSH expression is associated with increased immune suppression and decreased immune infiltration [10, 39].

**Fig. 9:**
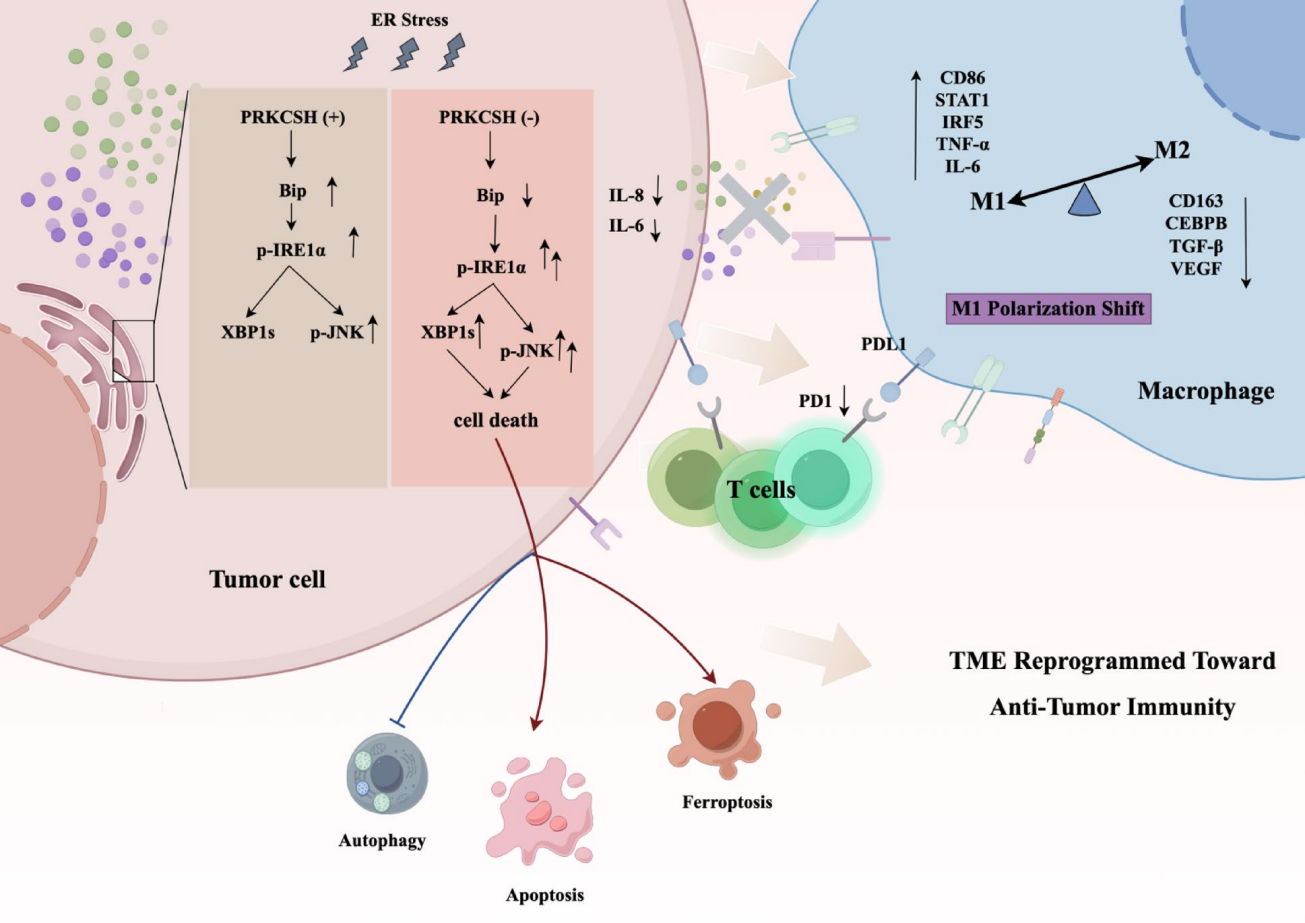
Proposed model illustrating the impact of PRKCSH deficiency on ER stress signaling, macrophage polarization, and tumor immune microenvironment remodeling

Together, our results establish PRKCSH as a complex modulator of immune escape and tumor cell survival that combines cytokine-mediated immune modulation and ER stress adaptation to promote a tumor-permissive environment. The loss of PRKCSH disarrays this equilibrium by increasing ER stress signaling through hyperactivation of the IRE1α–XBP1–JNK axis, as shown in Fig. 9. This results in decreased secretion of IL-6 and IL-8, impaired autophagy, and heightened vulnerability to ferroptosis and apoptosis in tumor cells. By increasing M1 macrophage polarization and decreasing M2-driven immunosuppression, these modifications aid in reshaping the tumor microenvironment and eventually fostering an immune response that fights the tumor.

Targeting PRKCSH may have several therapeutic advantages, including reestablishing immune surveillance through M1 reactivation, making tumors more susceptible to apoptosis and ferroptosis, and disrupting stress-adaptive systems that promote tumor survival. Moreover, the increased IRE1α activation in PRKCSH-KO tumors cells implies that ER stress inducers and PRKCSH inhibition may work in combination to improve therapeutic efficacy, especially in tumors that overexpress PRKCSH. There are still a few restrictions even though this study shows PRKCSH to be a crucial modulator of tumor immunity and stress reactions. In our zebrafish assays, the classification of macrophages into M1- and M2-like states by morphometric parameters (Feret diameter, aspect ratio, circularity) is an indirect proxy adapted from mammalian systems and may not fully capture the complexity of macrophage activation in vivo. Future in-vivo work should incorporate more direct phenotyping (e.g., reporter lines or transcript/protein markers) and extend beyond zebrafish to murine models. In addition, examining other tumor types will help determine whether PRKCSH effects are context-dependent, and further studies are needed to delineate how PRKCSH regulates cytokine secretion, particularly IL-6 and IL-8.

In conclusion, this study shows that PRKCSH coordinates cytokine regulation, immune cell reprogramming, and ER stress signaling to control tumor–immune dynamics in lung adenocarcinoma. PRKCSH’s function as an immune modulator and stress rheostat is highlighted by the suggested model (Fig. 9). To improve outcomes in lung cancer, future research should examine its potential as a therapeutic target, either alone or in conjunction with immune checkpoint blockade, ER stress modulators, or ferroptosis inducers.

Under ER stress, *PRKCSH*-expressing tumor cells (PRKCSH [+]) exhibit moderate activation of the IRE1α–XBP1–JNK pathway and secrete IL-6 and IL-8, promoting tumor survival and supporting M2 macrophage polarization. In contrast, *PRKCSH*-knockout tumor cells (PRKCSH [−]) show hyperactivation of the IRE1α axis, increased XBP1s and p-JNK levels, and enhanced cell death via apoptosis and ferroptosis, alongside reduced autophagy. The resulting decrease in IL-6 and IL-8 dampens M2-skewing signals. Conditioned medium from *PRKCSH*-KO cells shifts macrophages toward a pro-inflammatory M1 phenotype, with increased expression of CD86, STAT1, IRF5, TNF-α, and IL-6, and reduced M2-associated markers including CD163, CEBPB, TGF-β, and VEGF. These changes collectively reprogram the tumor microenvironment (TME) toward anti-tumor immunity.

## Supplementary Information

Supplementary Material 1.

Supplementary Material 2.

## Data Availability

No datasets were generated or analysed during the current study.

## References

[1] Chen OI, et al. A complex scenario and underestimated challenge: the tumor Microenvironment, ER Stress, and cancer treatment. Curr Med Chem. 2018;25(21):2465–502. 29345569 10.2174/0929867325666180117110259

[2] Hong SH, et al. Molecular crosstalk between ferroptosis and apoptosis: emerging role of ER stress-induced p53-independent PUMA expression. Oncotarget. 2017;8(70):115164–78. 29383150 10.18632/oncotarget.23046 PMC5777762

[3] Adams CJ, et al. Structure and molecular mechanism of ER stress signaling by the unfolded protein response signal activator IRE1. Front Mol Biosci. 2019;6:11. 30931312 10.3389/fmolb.2019.00011 PMC6423427

[4] Rubio-Patino C, et al. Reshaping the immune tumor microenvironment through IRE1 signaling. Trends Mol Med. 2018;24(7):607–14. 29804923 10.1016/j.molmed.2018.05.005

[5] Cubillos-Ruiz JR, Bettigole SE, Glimcher LH. Molecular pathways: immunosuppressive roles of IRE1alpha-XBP1 signaling in dendritic cells of the tumor microenvironment. Clin Cancer Res. 2016;22(9):2121–6. 26979393 10.1158/1078-0432.CCR-15-1570 PMC4854763

[6] Pelizzari-Raymundo D, et al. IRE1 RNase controls CD95-mediated cell death. EMBO Rep. 2024;25(4):1792–813. 38383861 10.1038/s44319-024-00095-9 PMC11014915

[7] Flores-Santibanez F, et al. Nuanced role for dendritic cell intrinsic IRE1 RNase in the regulation of antitumor adaptive immunity. Front Immunol. 2023;14:1209588. 37346037 10.3389/fimmu.2023.1209588 PMC10279875

[8] Shin GC, et al. PRKCSH contributes to tumorigenesis by selective boosting of IRE1 signaling pathway. Nat Commun. 2019;10(1):3185. 31320625 10.1038/s41467-019-11019-w PMC6639383

[9] Khaodee W, et al. Transcriptomic analysis of glucosidase II beta subunit (GluIIss) knockout A549 cells reveals its roles in regulation of cell adhesion molecules (CAMs) and anti-tumor immunity. BMC Genomics. 2024;25(1):82. 38245670 10.1186/s12864-023-09888-z PMC10799456

[10] Cressey R, et al. Navigating prkcsh’s impact on cancer: from N-linked glycosylation to death pathway and anti-tumor immunity. Front Oncol. 2024;14:1378694. 38571496 10.3389/fonc.2024.1378694 PMC10987803

[11] Lei R, et al. Potential role of PRKCSH in lung cancer: bioinformatics analysis and a case study of nano ZnO. Nanoscale. 2022;14(12):4495–510. 35254362 10.1039/d1nr08133k

[12] Khaodee W, et al. Knockout of glucosidase II beta subunit inhibits growth and metastatic potential of lung cancer cells by inhibiting receptor tyrosine kinase activities. Sci Rep. 2019;9(1):10394. 31316108 10.1038/s41598-019-46701-y PMC6637200

[13] Khaodee W, et al. Glucosidase II beta subunit (GluIIbeta) plays a role in autophagy and apoptosis regulation in lung carcinoma cells in a p53-dependent manner. Cell Oncol (Dordr). 2017;40(6):579–91. 28929344 10.1007/s13402-017-0349-1 PMC13001552

[14] Suradej B, et al. Glucosidase II exhibits similarity to the p53 tumor suppressor in regards to structure and behavior in response to stress signals: a potential novel cancer biomarker. Oncol Rep. 2013;30(5):2511–9. 24008518 10.3892/or.2013.2721

[15] Shin GC, et al. PRKCSH contributes to TNFSF resistance by extending IGF1R half-life and activation in lung cancer. Exp Mol Med. 2024;56(1):192–209. 38200153 10.1038/s12276-023-01147-1 PMC10834952

[16] Cao J, Liu C. Mechanistic studies of tumor-associated macrophage immunotherapy. Front Immunol. 2024;15:1476565. 39403370 10.3389/fimmu.2024.1476565 PMC11472702

[17] Batista A, et al. IRE1alpha regulates macrophage polarization, PD-L1 expression, and tumor survival. PLoS Biol. 2020;18(6):e3000687. 32520957 10.1371/journal.pbio.3000687 PMC7307794

[18] Fang P, et al. IRE1alpha-XBP1 signaling pathway regulates IL-6 expression and promotes progression of hepatocellular carcinoma. Oncol Lett. 2018;16(4):4729–36. 30214606 10.3892/ol.2018.9176 PMC6126152

[19] Hull-Ryde EA et al. IRE1alpha is a therapeutic target for cystic fibrosis airway inflammation. Int J Mol Sci. 2021;22(6):3060. 10.3390/ijms22063063 PMC8002512 33802742

[20] Jong KXJ, et al. IL-8 and PI3K pathway influence the susceptibility of TRAIL-sensitive colorectal cancer cells to TRAIL-induced cell death. Mol Biol Rep. 2024;51(1):978. 39269555 10.1007/s11033-024-09895-7

[21] Liu Z, et al. BEST: a web application for comprehensive biomarker exploration on large-scale data in solid tumors. J Big Data. 2023;10:165.

[22] Newman AM, et al. Robust enumeration of cell subsets from tissue expression profiles. Nat Methods. 2015;12(5):453–7. 25822800 10.1038/nmeth.3337 PMC4739640

[23] Huang C, et al. ScCancerExplorer: a comprehensive database for interactively exploring single-cell multi-omics data of human pan-cancer. Nucleic Acids Res. 2025;53(D1):D1526–35. 39558175 10.1093/nar/gkae1100 PMC11701644

[24] Chen X, et al. Benefits of zebrafish xenograft models in cancer research. Front Cell Dev Biol. 2021;9:616551. 33644052 10.3389/fcell.2021.616551 PMC7905065

[25] Rostam HM, et al. Image based machine learning for identification of macrophage subsets. Sci Rep. 2017;7(1):3521. 28615717 10.1038/s41598-017-03780-z PMC5471192

[26] McWhorter FY, et al. Modulation of macrophage phenotype by cell shape. Proc Natl Acad Sci U S A. 2013;110(43):17253–8. 24101477 10.1073/pnas.1308887110 PMC3808615

[27] Zou Z, et al. Tumor-associated macrophage polarization in the inflammatory tumor microenvironment. Front Oncol. 2023;13:1103149. 36816959 10.3389/fonc.2023.1103149 PMC9934926

[28] Gupta S, et al. *IL*-6 augments *IL*-4-induced polarization of primary human macrophages through synergy of STAT3, STAT6 and BATF transcription factors. Oncoimmunology. 2018;7(10):e1494110. 30288360 10.1080/2162402X.2018.1494110 PMC6169572

[29] Chen L, et al. *IL*-6 influences the polarization of macrophages and the formation and growth of colorectal tumor. Oncotarget. 2018;9(25):17443–54. 29707119 10.18632/oncotarget.24734 PMC5915127

[30] Tsukamoto H, et al. Immune-suppressive effects of interleukin-6 on T-cell-mediated anti-tumor immunity. Cancer Sci. 2018;109(3):523–30. 29090850 10.1111/cas.13433 PMC5834784

[31] Teijeira A, et al. IL8, Neutrophils, and NETs in a collusion against cancer immunity and immunotherapy. Clin Cancer Res. 2021;27(9):2383–93. 33376096 10.1158/1078-0432.CCR-20-1319

[32] Xiao P, et al. Neurotensin/*IL*-8 pathway orchestrates local inflammatory response and tumor invasion by inducing M2 polarization of Tumor-Associated macrophages and epithelial-mesenchymal transition of hepatocellular carcinoma cells. Oncoimmunology. 2018;7(7):e1440166. 29900041 10.1080/2162402X.2018.1440166 PMC5993481

[33] Jiang M, et al. Dual inhibition of endoplasmic reticulum stress and oxidation stress manipulates the polarization of macrophages under hypoxia to sensitize immunotherapy. ACS Nano. 2021;15(9):14522–34. 34414762 10.1021/acsnano.1c04068

[34] Ayaub EA, et al. IL-6 mediates ER expansion during hyperpolarization of alternatively activated macrophages. Immunol Cell Biol. 2019;97(2):203–17. 30298952 10.1111/imcb.12212 PMC7379543

[35] Mahadevan NR, et al. Transmission of Endoplasmic reticulum stress and pro-inflammation from tumor cells to myeloid cells. Proc Natl Acad Sci U S A. 2011;108(16):6561–6. 21464300 10.1073/pnas.1008942108 PMC3081038

[36] Rahal OM, et al. Blocking Interleukin (IL)4- and IL13-Mediated phosphorylation of STAT6 (Tyr641) decreases M2 polarization of macrophages and protects against Macrophage-Mediated radioresistance of inflammatory breast cancer. Int J Radiat Oncol Biol Phys. 2018;100(4):1034–43. 29485045 10.1016/j.ijrobp.2017.11.043

[37] Niu X, et al. Autophagy in cancer development, immune evasion, and drug resistance. Drug Resist Updat. 2025;78:101170. 39603146 10.1016/j.drup.2024.101170

[38] Yamamoto K, et al. Autophagy as a critical driver of metabolic adaptation, therapeutic resistance, and immune evasion of cancer. Curr Opin Biotechnol. 2023;84:103012. 39492353 10.1016/j.copbio.2023.103012

[39] Wang Q, et al. PRKCSH serves as a potential immunological and prognostic biomarker in pan-cancer. Sci Rep. 2024;14(1):1778. 38245572 10.1038/s41598-024-52153-w PMC10799934

