# Supplementary material for: PRKCSH deficiency promotes an anti-tumor immune microenvironment via UPR activation and M1 macrophage polarization
Source: Cancer Cell Int. 2025 Dec 5;26:4. doi: 10.1186/s12935-025-04104-2 (PMC12797428; doi:10.1186/s12935-025-04104-2)
Supplement: Supplementary file 1 — Supplementary Material 1. [file 12935_2025_4104_MOESM1_ESM.docx]

**Supplementary Table 1: Lists the primers and probes used for the quantitative real-time PCR (qRT-PCR) experiments.**

| **Name of Primers/ Probes** | **Sequence (5' to 3')** |
| --- | --- |
| GAPDHForward | CATCCTGGGCTACACTGAGC |
| GAPDH Reverse | AAAGTGGTCGTTGAGGGCAA |
| VEGF (human)Forward | CCTTGCCTTGCTGCTCTACCTC |
| VEGF (human) Reverse | GATGATTCTGCCCTCCTCCTTCTG |
| GAPDH (human)Forward | CATCCTGGGCTACACTGAGC |
| GAPDH (human) Reverse | AAAGTGGTCGTTGAGGGCAA |
| TGF-β (human )Forward | GCAACAATTCCTGGCGATACCTC |
| TGF-β (human ) Reverse | CCTCCACGGCTCAACCACTG |
| IL-6 (human)Forward | ATGAACTCCTTCTCCACAAGCGC |
| IL-6 (human) Reverse | GGGAAGGCAGCAGGCAACAC |
| TNF-a(human)Forward | GAGCACTGAAAGCATGATCC |
| TNF-a(human) Reverse | CGAGAAGATGATCTGACTGCC |
| IRF5 (human)Forward | CTCGACTGCTGCTGGAGATGTTC |
| IRF5 (human) Reverse | CGGTCTTTGAGGTCTGGGTTTGAG |
| CEBPB (human)Forward | TACTACGAGGCGGACTGCTTGG |
| CEBPB (human)- Reverse | AGGTACGGGCTGAAGTCGATGG |
| stat1(human)Forward | ATGCTGGCACCAGAACGAATGAG |
| stat1(human) Reverse | TCACCACAACGGGCAGAGAGG |
| stat6(human)Forward | CGGAGCCACTACAAGCCTGAAC |
| stat6(human) Reverse | GGTCCCTTTCCACGGTCATCTTG |
| INF-γ（human）Forward | TGACTTGAATGTCCAACGCAAAGC |
| INF-γ（human）Reverse | CGACCTCGAAACAGCATCTGACTC |
| Granzyme B（human）Forward | GTGCGGTGGCTTCCTGATACG |
| Granzyme B（human）Reverse | TGCTGGGTCGGCTCCTGTTC |
| PD1（human）Forward | CCAGGATGGTTCTTAGACTCCC |
| PD1（human）Reverse | TTTAGCACGAAGCTCTCCGAT |
| VEGF (zebrafish) Forward | ATCCCGTCCTGTGTGGTTCTC |
| VEGF (zebrafish) Reverse | GACCCGCAGCACCTCCATAG |
| GAPDH (zebrafish) Forward | CATCCTGGGCTACACTGAGC |
| GAPDH (zebrafish) Reverse | AAAGTGGTCGTTGAGGGCAA |
| TGF-β (zebrafish ) Forward | CCAGCAGAGCACGGATAAGTTC |
| TGF-β (zebrafish ) Reverse | AATGTACGGCTTCACCATCATATCTG |
| IL-6 (zebrafish) Forward | TCTGCTACACTGGCTACACTCTTC |
| IL-6 (zebrafish) Reverse | CTGAATCTGAAGAGTCAATACCGCCAGAAT |
| TNF-a (zebrafish) Forward | GCCTTTGTGCCGCTGCTG |
| TNF-a (zebrafish) Reverse | TGGAAGTGAAATTGCCTTGTGAAATG |
| IRF5 (zebrafish) Forward | CCCACCACAACCCTCCAATGAAC |
| IRF5 (zebrafish) Reverse | GCCAGATGAGCGACCATAGAAGC |
| CEBPB (zebrafish) Forward | TCAAGCGGGAAAGGCAAGAAGC |
| CEBPB (zebrafish) Reverse | TTGCGGACGGCGAGATTGTTC |
| stat1(zebrafish) Forward | GCTCTCCGCTGTTTCACTTCCTG |
| stat1(zebrafish) Reverse | GCTTGTCAGTGGGCTTGGTGTAG |
| stat6(zebrafish) Forward | ACTGTACGCTATCTGCTGGGAGAC |
| stat6(zebrafish) Reverse | TGCTTGTGCCTCGGTGATAATCTG |

| **Antibody** | **Manufacturer** | **Cat no.** | **Dilution used** | **species** | **Observed band size** |
| --- | --- | --- | --- | --- | --- |
| Anti-BAX Antibody | Huabio | ER0907 | 1:3500 | Rabbit P | 21kDa |
| Anti-Bcl-2 Antibody [JF104-8] | Huabio | ET1702-53 | 1:1500 | R Rabbit M | 26kDa |
| COX-2(29):sc-19999 | Santa cruz | sc-19999 | 1:500 | Mouse M | 70-72kDa |
| Anti-GPX4 Antibody [JU11-31] | Huabio | ET1706-45 | 1:10000 | R Rabbit M | 22kDa |
| Rb mAb to GRP78 BIP[EPR404d27] | abcam | ab108613 | 1:5000 | Rabbit M | 78kDa |
| IRE1α（14C10）Rabbit mAb | Cell Signaling Technology | #3294T | 1:1000 | Rabbit M | 130kDa |
| Anti-IRE1(phospho S724) antibody [EPR23107-16] | Abcam | ab243665 | 1:1000 | Rabbit M | 130KDa |
| Anti-Glucosidase 2 subunit beta antibody [EPR8047] | Abcam | ab129098 | 1:10000 | Rabbit M | 80kDa |
| Rb mAb to xbp1[EPR4086] | abcam | ab109221 | 1:5000 | Rabbit M | 29kDa |
| MS mAb to XBP1[143F] | Abcam | ab241571 | 1:1000 | Mouse M | 54kDa |
| Anti-JNK1+JNK2+JNK3 Antibody [SA43-06] | Huabio | ET1601-28 | 1:2000 | R Rabbit M | 48,53kDa |
| Anti-Phospho-JNK1/2/3(T183+T183+T221) Antibody [ST500] | Huabio | ET1609-42 | 1:1500 | R Rabbit M | 48,53kDa |
| Anti-LC3B Antibody [JJ090-6] | Huabio | ET1701-65 | 1:1000 | R Rabbit M | 14/16kDa |
| β-Actin (13E5) Rabbit mAb #4970 | Cell Signaling Technology | #4970T | 1:1000 | R Rabbit M | 45kDa |
|  |  |  |  |  |  |
| **Flow Cytometry Antibody** | **Manufacturer** | **Cat no.** | **Dilution used** | **species** | **The target species** |
| BD Pharmingen™ APC Annexin V | BD Pharmingen | 550474 | 5ul |  | Human |
| BD Pharmingen™ 7-AAD | BD Pharmingen | 559925 | 5ul |  | Human |
| BD Horizon™ Fixable Viability Stain 780 | BD Pharmingen | 565388 | 1:1000 |  | Human |
| PE/Cyanine5.5 Anti-Human CD45 Antibody[HI30] | Elabscience | E-AB-F1137I | 5ul | Mouse | Human |
| APC-Cy7 Mouse Anti-Human CD45(2D1) | BD Pharmingen | 561863 | 5ul | Mouse | Human |
| CD11b Monoclonal Antibody (ICRF44), FITC, | eBioscience™ | 11-0118-42 - | 5ul | Mouse | Human |
| Alexa Fluor 647 Mouse Anti-Human CD68(Y1/82A) | BD Pharmingen | 562111 | 5ul | Mouse | Human |
| PE Mouse Anti-Human CD86(2331 (FUN-1)) | BD Pharmingen | 557344 | 5ul | Mouse | Human |
| CD163 Monoclonal Antibody (eBioGHI/61 ( PE-Cyanine7)) | eBioscience™ | 25-1639-42 | 5ul | Mouse | Human |
| FITC Plus Anti-Human CD68 Rabbit Recombinant Antibody | proteintech | FITC-98242 | 5ul | Rabbit | Human |

**Supplementary Table 2**: lists the antibodies used in the study, along with their manufacturer, catalog number, recommended dilution, actual dilution applied, species, and observed band size.
