# Supplementary material for: PRKCSH deficiency promotes an anti-tumor immune microenvironment via UPR activation and M1 macrophage polarization
Source: Cancer Cell Int. 2025 Dec 5;26:4. doi: 10.1186/s12935-025-04104-2 (PMC12797428; doi:10.1186/s12935-025-04104-2)
Supplement: Supplementary file 2 — Supplementary Material 2. [file 12935_2025_4104_MOESM2_ESM.zip › PRKCSH-KO sequencing/PRKCSH Full length of the genome(FROM NCBI).docx]

**PRKCSH Full length of the genome(FROM NCBI)**

**Primer-F 152：GAGGCTGTTAATCCCTTTGGTCT**

**Primer-R 782：TCTACGATGAGTCCAAGCCTTTC**

**gRNA ACCCATGTGCTGGGCCGTGG**


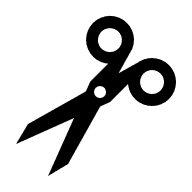

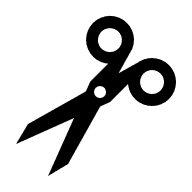
CTTTCTGCAGCAGGAACCGCGGCTGCTGGACAAGAGGGGTGCGGTGGATACTGACCTTTGCTCCGGCCTCGTGTAGGTGTGAACGAGCGGGTGGGAGGGCACCTCAGTTTCTTACAGGGGGCAACCGGAGGGTGCATGTGTGGGTGTGGACGGCGGAGGCTGTTAATCCCTTTGGTCTGTTGGCGGGAATTGGGTCACAATTGGGCACAGGGAGCGGAGGTTTTGAGATCTTGAGATCCTCGCCATTGATTTGAGCAAAGTGAGACTGGCCAGGATTGGGGAGATCGCTGCCCCTCGCCCCCATATCGGAAACAAAGTGAGGCCTCATTGCCTGGAAGCGATGGAGGAATCCAATTGGCTGGAAGTAGCCCTCTCCCACTGACCGGGATCCGCTTTCTTCCTGCAGCGTGAAGACACAGCGCATCTCCCCGCTGTAGGCTTCCTCCCACAGAACCCGTTTCGGGCCTCAGAGCGTCTGGTGAGATGCTGTTGCCGCTGCTGCTGCTGCTACCCATGTGCTGGGCCGTGGAGGTCAAGAGGCCCCGGGGCGTCTCCCTCACCAGTGAGTCCTCCTGTTCACCCTCCCGCCAGGCTGGAGGTGGGAGGGGCCAACATTGGGCCTTAGGGATAGGCATTTACAGCCTTTTGACTCAGTTTCCCGGTAGTGCTCCGCTGGTGGGGATGGGAGGACAGAGGTGGTATTTGGTGGAGAAGGCGCTTACCTGCCCTGGGCTGAGCTTCCTGTACCCCGCAGATCATCACTTCTACGATGAGTCCAAGCCTTTCACCTGCCTGGACGGTTCGGCCACCATCCCATTTGATCAGGTCAACGATGACTATTGCGACTGCAAAGATGGCTCTGACGAGCCAGGTGAGCCTTTTCTCTGTTCATCCATCAGATGTTTATTGAACACTGCTCAGTGCCAGGCCCTGTCTGTGTGACCAAGATACTACCTCTGCTGGCTCCCCTTTGAGTGGGCAGAAACAAGCTCAGAAGTCAGTGGTCAGTGTTATGGCCCCGGGCAGCTGGAGGAGCCCAGAGTCGTCCTCCTCCCAAGCCTCGGGGTCAGAGAAGGCTCCTCCAAGGAGGTGAGGTTTCAGTTGAATCCCGAAGGTGCTGCAGATGGAGGGGTCAACACGGGCAAACCCCTAGAAGTGGGAGACAGGGTGGGGCCAAGAGCAATCCAGCAGGAGACAGAGGTGAAGGGAGACCCTGGCTTTTTGTTTTTGTGTGTTTTGAAGAGACAGGGTCTTGCTTTGTTGCTGAGGCTGGAGTACAGTGGCATGATCGTAGCTCACTGCAGCCTGGAACTCTTGGGCTCAAGTCATCCTCCTGCCTTGGCCTCCCAAAGTGCTGGGGTTACAGGTGTGAGCCACCGTGCCTGGATGAGACCGTGTCTTTGGTTTTGTTTTTGTTTTGAGACGGAGTCTTGCTCTGTTTCCCAGGCTGGAGTGCAGTGGCGCCATCTCGGCTCACTGCAAGCTCCGCCTCCCGGGTTCACACCGTTCTCCTGCCTCAGCCTCCTGAGTAGCTGGGACTACAGGCACCCACCACCACGCCCAGCTAGTTTTTTTTTGTATTTTTAGTAGATACAGGGTTTCACTGTGTTAGCCAGGATGGTCTCGATCTCGTCACCTTGTGATCCACCCGCCTCGGCCTCCCAAAGTGTTGGGATTACAGGCGTGAGCCACCGTACCTGGCCTTGTTTTGTTTTGTTTTTAATTTTTTTTTTTGAGATGGAGTTTCACTCTTATCGCCCAGGCTGGAGTGCAGTGGCATGATCTTGGCTCACTGCAGTCTCCACCTCCCGGGTTCAAGCGATTCTCCTGCCTCAGCCTCCTGAGTAGCTGGGATTACAGGCACATGCCACCACATCCGGCTAATTTTTCGTATTTTTAGTAGAGATGGAGTTTCATCGTGTTGGCCAGGCTGGTCTCAAACTCATCACCTCAGGTGACCCACCCGCCTCGGCCTCCCAAAGTGCTGGGATTACAGGTGTGAGCCCCCGTGCCAGGTGGAGACCCTGTTTTGAAGGGCCTCAGTGAGAAGAGGCAGGAGGCGGCACTGGAGAGCTTTATTTCGAGCCCTGGCTGTGCAGTGTACAACTCTTTCCTTCCTTTGGTTTCCTTTCCTTTCTTGTGCTGTGTGCAGTGTGGGTCAGGGGCTCTTATCTGTGGATGGATGGGACATGTCTGTCCCTGAGCTGACTCCGAAAACCTTCCCTCCCTTCTTCCTCACAGGCACGGCTGCCTGTCCTAATGGCAGCTTCCACTGCACCAACACTGGCTATAAGCCCCTGTATATCCCCTCCAACCGGGTCAACGATGGTGTTTGTGGTAAGTGAAGATGCACCAGGATTCTGGAAAGGTGGTAGAGGGAGGGAGGGAGGAGGCACTGCCAGGTCTGATCTTGGCTTCTGCCTCTGCCACAGACTGCTGCGATGGAACAGACGAGTACAACAGCGGCGTCATCTGTGAGAACACCTGCAAGTACGTGGGTGACAGTACCCCTCCCATCACCCCACCCCAAGACTTTGCCTGGCTCCACCCAGTGAATCGGGCCCACTCTCTCTTCTGCTTTTCTGTATCGGGTTCTCTGTCTGTGCCAGGTGCCTTGTGAATCCTAATCCCCACCTTTCCCTCTAGTCTGGAAGCAGGTAGGATTGTTTCCCGATTCAACAGATGGGGGAACTCAAGTTTAGAAAGGGAAATGACTTCCCCAGGGTCACATAGGAGATAGCAGAGCCGGCACTTGGACTCTAAACTTTCCATTGTCACGCTCTGCAGCAGGTCCTGAAAACCGAAACACCCACAGGCAGGTAAAGTATACAACGGAAGGAGGCCAGGCGCGGTGGCTCACACCTGTAATCCCAGCACTTTGGGAGGCTGAGGCAGGCGGATCACGAGGTCAGGAGATCGAGACCATCCTGGCTGACATGGTGAAACTCCGTCTCACTAAAAATACAAAAAATTAGCTGGGTGTGGTGGCGGGCGCCTGTAGTCCCAGCTACTCGGTGGGCTGAGGCAGGTGAGTGGCGTGAACACGGGAGGCAGAGGTTGCAGTGAGCCGAGATCGCACCACTGCAATCCAGCCTGGGCAACAGAGTGAGACTCCGTCTCAAAAAAAAAGCAAAAAAAAAAAAAGCATACAACAGAAGGAGGCCAAGTATGAGGCAATACGGAGGGGTGGGGATTGTGGCATCTAGGGGAACAGCTGTTCTCAATTCCTGCCAGCTGATCCTTTTGTAGTTCATAGGCATGATGATTTATTTTTATTTATTTATATTTTTTGAGACAGGGTCTCACTCTGTCGCCCAGGCTGGAGTGCAGTGGCGTGATCTTGGCTCACTGCAACCTCTGCCCTTCGGGTTCAAGCAATTCTCACACCTCAGCCTCCCAAGTAGCTGGGAATACAGGCGTACCCCCACCACGCCTGGCTAACTTTTGTATTTTTGGTAGAGATGGTTTCACTATGTTGGCCAGGCTGGCCTCGAACGCCTGACCTCAGGTGATCTGCTCGCCTCAGCCTCCCAAAGTGCTAAGCCGCCGCGCTTGGCCACGATGATTAGGTTTTTATGCACGTGTGAGAGATACGCCTCCCTTAAACCTTGTTGCGACCTTGGCGCATTACCTGTCTGATATGAAAAAATAAGACCCGGCTCATGGTGGCTCCTGCCTGTAATCCCAGCATTTTGGGAGGCTGAGGCAGGAAGACTGCTTGAGCCCAGGAGTTTGAGACCAGCCTGGGCAAAATAGCGATACCCTATCTCTATAAAAAGTAAAAAAAAAAAATTAAAAAGTAAAAATAAAAATTGGAAATTATAATTTAAAAAATTACTGGTTGGGCATGTGGCTCACACCTGTAATCCCAGCACTTTGGGAGGCTAAGGCGAGAGGATCACCTTAACCCAGGAGTTTGAGACCAGCCTGGGCAACACAGTGAGACCCGTCTCAGAAAAATTTTTCTTTTCTTTTTTTTTTTTTTTTTTTTTTTTTTTGTGAGACAGAGTCTCACTCTGTTGCCCAGGCTGGATGGAGTGCAGTGGCGCGATCTCGGCTCACTCTAACCTCTGCCTCCCGGGTTCAAGCGATTCTTCTGCCTTAGCCTCCTGAGTAGCTGGGACTACAGGCATCCACCACCACACCCGGCTAAAATTTGTATTTTTAGTAGAGACGGTGTTTCACAATATTGGCCAGGCTGGTCTCGAACTCCTGACCTCATGATCCACCTGCCTTGGCCTCCCAAAGTGCGGGGATTACAGATGTGAGCCACCATGCCCGGCTGAAAATTTGTTAAAAAATTAGCCGGGTGTGTTGGTGCGTGTCTGTAGTCGCAGGTACTCAGGAGGCTGAGGCAGGAAGATTGCTTGGGCCTGGGAAGCAGAGGTTGCAGTGAGCTAAAAAGTAAAAAAAAAACAAAAAAAACACAAAGTCCTATCAGTTGTTGGCAGATGGTGTAAAACTCTCTGGATTCCACATCTTTTAAATAGAAGGTGGATTGAACACAACCAACACACTTCTTTTTTTTTTTGAGACGGAGTCTCACTCTGTCGCCCAGGCTGGAGTGCAGTGGTGTGATCTCCGCTCACTGCAAGCTCCGCTTCCCGAGTTCATGCCATTCTCCTGCCTCAGCCTCCCGAGTAGCTGGGACTACAGGCACCTGCCGCTACACCTGGCTAATTTTTTTTTTTGTATTTTTAGTAGAGATGGGGTTTCACCGTGTTAGCCCGGATGGTCTCGATCTCCTGACCTTGTGATCTGCCCGCCTCGGTCTCCCAAAGTGCTGGGATTACAGGTGTGAGCCACCGCCCCTGGCCCACACTTTTTTTTGAGTTAGAGTCTCGCTCTGTTGCCCAGGCTGGAGTGCAGTGGCATGATCTTTGCTTACCCCAACCTCCACATCCGGGTTCAAGTGATTCTCTTGCCTCAGCCTCCCGTGTAGCTGGGATTACACCGTGTAGCTGGGCGCCACCACGCCCAGCTAATTTTTGCATTTTCAGTAGAGACAGGGTTTCACCATCTTGGCCAGGCTGGTCTCGAACTCCTGACCTCAGGTGATCCGCCCGCCTTGGCCTCCCAAAGTGCTGGGATTACAGGCATGAGCCACCACGCCCAGCCAACCAACACACTTAGTAACCAGATTGTCTTTTTGTCTGGGAGTCATGATTTCTGTTCTGTCTTCTGTGGTTTTTTTATTTGTTTTAATTATGTGTAAAGACAGGGTCTTTATATGTTGCCCAGGCTGGTTGCGAACTCCTGGGCTCAAGTGATCCTCCTGCCTTGGCCTTTCAAACTGCTGGGATTACAGGCATGAACCACTGCACCCAGCTGATCTCCTGTCTCTTGAGAGTTGATATCTAGCTCCACCTCCTGGCACTCCCTAGTCCCTTCCCTGACCCCAGGGGCTTACAGAACCCACTGTTCCTCTTTCAAGGACAGGAATGAAGGATGCTGATGTTGAGAGAACCTGGGAGGAAGGCTTGCTCTCATCTTTCCCAGCATGGCTTGGTGGGGGGCCACAGCTGGATTGAGCTATTTTGGAAGAGGCAGACCTGGTGGATCCTAAGTGCCCCACTGGTGGTGCCTGTGTGTCTCCGCACCGCAGAGAGAAGGGCCGTAAGGAGAGAGAGTCCCTGCAGCAGATGGCCGAGGTCACCCGCGAAGGGTTCCGTCTGAAGAAGATCCTTATTGAGGACTGGAAGAAGGCACGGGAGGAGAAGCAGGTAAGGAACCCGCGGGGGCTGCCCCAGGGTGATCTGGGCCTTGAGGCCTCATAGTGTGGGCTTGCAAGGCTGGGGAGGGGTTTGCCCCTGACTGCTGTTCTGGGGCAAGTGACTGCCTTTCGGAGCTTTGCTTTCTCAGTTGTGTGCTAGGCGTTTAGTCATTGGCTAAGTATAATAGTTCCCGAGGGGGAGGCTGTCAGCTTAGGTGTTTATCAGGGCCTCGGGCATCAGACTAAAGCAGGCTGGAAGGGCCTCTCTGCCTCCTGGGGATGGGAGGGTTGCTTCTCTGTACCCCGGATCTGTGGTGCCATTGCCTGTAAGATGGGCTGATGACCAGATGACTCCCCAGTTCTGATGTTAGAAAGATAACGTCAGCCAGTAGCAGCTGGATTAATTCCATAGGGTGGGTGCTTTGGGGTCCCTAGGTGTGTGTCTCTCCCTGCCTTGCCTGTGTACTGGGCAGGAGGCTGGAAACCGACCTGGATTTGCCTCAAGGCATTCATTGCTAGTGGGAGGGACAGCCAGCCAGGGCTGCTGGACAAAGGGAAATTCGGGGTAGAGACTTCTTGCCCAGTTCCTCTCCCTCTCTGCCTTGGGGGCAGGAGGCAAGGAAGGCTTCTTGGAGGAGGAGGCCTCTATCTCACATGAAATCTGTTGGGTAATTTGGAGTTAGCCAGGTAGAGTGTTCCAGGCTGAAGGAACAGCATGTTTGAAGAACCGGAGGCAGAAGAGCGTTTAGGGGTCTGAATGTTGTCAGGGCGACTGGATCCTAGTAAGATAGGGATGGGGCAAATGGGGCTGGAGAATCAGGCAGGGGCCTGATTACCTGGGCCCTTGCGGGCTACATTGAGGCGTTTGGGCTTCTCTCCAGAGGGCTACAGGGAACCCCAGCTCGGGAGAGAGACCCAGCTTGGTGTGTGTTTTGGAACATCTCCCTGCTGCCCTGTGGAGTAGAGGCAGGGAGGTAGTAGTCAATGAGGAGGAGGCAGAACAGAGGAGAGCTGGTCTCTTGCCTTCTGCCCACCCAGAAAAAGCTCATTGAGCTACAGGCTGGGAAGAAGTCTCTGGAAGACCAGGTGGAGATGCTGCGGACAGTGAAGGAGGAAGCTGAGAAGCCAGAGAGAGAGGCCAAAGAGCAGCACCAGAAGCTGTGGGAAGGTATGGCAGAAATGGCCAAGGACTCACCTTCAGTCCTGGGTGGGAGCAGGTCTGGGCCTAGGAGTCTCCCGCTCATTATCTGTTGTCAGTTTCAGCAGGTTTTTGATCATGCTGCCCATCGCCTTGTGTGCTTTGAGGTTCGCTTGGATTGTGGCATATGTCAGGAGCCTCCTTTTTAAAAATTGTTTAAATAATTTGTTTTTTGAAACAGCGTCTCCCTCTGCCACCCGGGCTGGAGTACAGTGGCGTGATCTCAGCTCACTGCAACCTCTGCCTCCCAGGTTCAAGCAATTCTTGTGCCTCAGCCTCCTGAGTAGCTGAGATTACAGGCACCTGCCACCACATTTAACTAATTGTTTTGTATTTTTAGTAGAGACGGGGTTTCACAATGTTGGCCAGGCTGGTCCTGAACTCCTGACCTCAAGTGATCCACCTGCCTTGGCCCCCCAGAGTGCTGGGATTACAGGCATGAGCCACCTCGCCCGGCCGCCTACTTCCTTTTAACATCAATGTAGATTTTGTTATATGACTTTAGCAGGACTTAGTTAATCCAGTGACCATTTTTCCTATCAGGTGTTTGGTATTTGTCTTCACTAATTGTAAGAGCTTTTTTGTGGCTGGGCATGGTGGCTCACGCCTGTAATCCCAGCAGTTTGGGAGGCCGAGACGGGCAGATCACCTGAGGTCAGGAGTTCGAGACCACCCTGGCCAACATGGTGAAACGCCATCTCTACTAAAAGAAAAAATTATGGCCGGGCACAGTGGCTCATGCCTGTAATCCCAGCACTTTGGGAGGCTGAGGCAGGCAGATCACGAGGTCAGGAGATTGAGACCATCTTAGCCAACATGGTGAAACCCATCTCTACTAAAAATACAAAAATCAGCTGGACATGGTGGTGTGTGCCTGTAATCCCAGCTACTCGGGAGGCTAAGGCAGGAGAATTGCTTGAACCAGGAAGTTGGAGGTTTTCAGTGAGCTGAGATTGCGCCCCTATATTCCAGCCTGGCAACAGAGTGAGACTCCGTCTCAAAAAAAAAAAAAAAATTAGCCTGGCATGGTGGCATCTGCCTGTGGTCCCAGCTACTTGGGAGGCTGAGGCAGGAGAATCGCTGGAACCCGGGAGACAGAGGTTGCAGTGAGCTGAGATCTCGCCACTGCACTCCAGCCTGGGTGACAAAGCAATACTTCATCTAAAAAAAATAATAAAGAGCTTTTTCTATCTGGGGTTTGGCCCTTTGTCTGCAACTGAAGATTTTTTTTTAGTTTATTTATTTTTGAGATGGAGTCTCACTCTGTCGCCTAGGCTGAAGTGCCAATGGCCCGATCTCTGCTCACTGCAACCTCTACCTCCTGGGTTCAAGTGATTCTCCTGCCTAGGCTTCCTGAGTAGCTGGGATTACAGGCACCTGCCACCACAGCCGGCTAATTTTTGTAGTTTTAGTAGAGACGGGGTTTCACCGTGTTGGCCAGGCTGGTCTTGAACTCCTGACCTCAGGTGGCCTGCTTTGGCCTCCCAACGTGTTGGGATTACAGGCATGAGCCACGGTGCCCAGCTCTGTCTGCAACTGAAGATATTATTTCCCTTCTTCATTTGTTTTTGGTGCTAGAGCCTGGTGTTTTTTGGTAAGGCCACATTGCATGTAGAAAGGGAATTCACTGTCTGGCTCTGTCTGCCCCTGAATCTTTGTCGTGTTGCAAGCTTCCTTTCACTTGGGTCTGGGCAAGCTCAGGCCTGATTTTCCTCAGGATGTGTTCTCAGTGTGGAGCTGTGGAATCAGAGGGAAGCGCCATGTTTTGACCCAGATTCCCAAAGGGTGGCGGGGCTGAGCCAGGGCTCCGAGGGTAGATGGCCTGGATTGGGCCCCAGGCTACACCTCTCACTGGCTAGGTGATTATGGGCAGGTCCCTTCACCCACAGAAAGGTCAGGGACCCCTCAGGCCCTCATCTGTGAGACTCAGAACAGTGTCAGCCGGGTCTCCTGGGACATGCGGGATGTCCTGGGGTGACCTAAGACTCTCTAGAGGAAATACCAGCTGTTTTTATTCTGTTTGTAGAGGACCAAGCTGGAGCTTGGTATACAGTGGGAACTGAATCCTGGTATACAGTAGGAAGTTAATCATTGGAGCCGCTGTTGTGGCTGCTTTCCGCTCTGCTTAGGTTAGAGTAGCGACAGACAAAGGAGGGACTTTTGGGACAGAGGCTCGGTCCCCTTCTGTCCGGCTGGCCCCTCTTCCTGGGAGCAGCCCTGGGGACCCCCAAGATGCCCCCAGGACCGGTCTCCCTCCCTGTATCCTCCCCATCTCCCAGCATCCTTGGCCATGAAGTCTCATTCCTGCCAGCCCTGCCCCGACATGCCTCTGCCAGGTCCTAAACCTAAAGTCTCAGTGGTCGCTCTCCTCTCCCCAACCCCACCTCCCACCTCTGACCGTCTGCGCACTCCATCTGCGTCTCCCCAGAATCTGCCTCTGCTCCGTGCTTCTTCCTCAGTCCCCAGCCTGGGCCAGCCCTTGTTATCACCTGGCTTCTGCCCTCGCCCCCACCTCTGTCCCAACAGTGGCCTGACAGGGCTGCCCTCTGCCACCTGGGTTCCCACCTCCCTGCTCCAGCCCCACCAGTGGCCCTCCTGCCTCTGCACTCCCCACCTTGTCCCGGTCTCCCCCAACACAACCAGGCTTCAACCATTCACCTTGCTCTTCGTCTGTTCTCCCCTGGTAAGGGCAGGGACCTTTGTCTCCTCCACAGTCGTGTCCTCAGGGTCCAGCATGGGGCCAGGCATATAGTAGGCGCTTGGTGGCAAACGACCCTGTGGGGTGCGGGGGCTGATGGGAGCCGGTGGGTGGGCAGGGGGTGACAGAGGTGGCTTCTTTACAGAGCAGCTGGCTGCTGCCAAGGCCCAACAGGAGCAGGAGCTGGCGGCTGATGCCTTCAAGGAGCTGGATGATGACATGGACGGGACGTGAGTGTCCCCTAGTTGGAGCTGCCCACCTTTCCGTGGGCCTGGGTTTCCCTCCCCGCCACCCTCGCCTCTAGAAACCAGCCAGATCCTCCTTGGGTTCCCCCGGCGTGGGGTCCAGGCTGATCCCAAGCCCCGTGTGACCCCGCCTCTTACTCGTGGATGGCCAGGTCCAGGATGCCCTGGGCGAGGAGATAGGGGGACCACCCTCTCCCCAGGAGCTGGGCACAGACCCTCAGCCTCAGGGAGTGTGTCTAGGCATCTAGTGATCGGATGGCTTTTCCCAGCCCCACTACGCTCTGGGGAAAGCCAGACCTGGGTTCGAATCCTGGCTGTGCCTCTGAGCTTGCTGGGTTACCTGGTGGAGGGTGGACTTTTGGGGGCTGAGAGGCACACGGGGAGCCTAGGAGATGTCAGGTGCCAAGGAGCAGGGGGCTTCAGAGGGACCGAATGGGCAAGGGTGGGGGTGGGCTTTGCTGGGTGGGGGACCAAGGCAGATGGCAGCTTCAATGTGCCGGGTCCTGGGGTGGAAGGAGGTGCCACCTGGGTGAGTTGGTAGAGAGGTCATCGGACATTTGGGGGTCCAGGTATCTCCCAGTCCCACCCCTTACTTTGAGCTCCTTTGGGGTCATGGAGTCCTGAGGAGGGGGCAGGAGGGTGGGGAGGCCCCTACACCTTGAGGTCCTGAAGCAAGTTCCAGGGTGGGGCCCTGCAGGGAAGAACAGGTGGGCCACATGGTGCCCCCAACTGAGAGCCACTGGGGCCTCACCCCTCCAGTCTGCTTCTGCCACGCCCCCGCAGGGTCTCGGTGACTGAGCTGCAGACTCACCCGGAGCTGGACACAGATGGGGATGGGGCGTTGTCAGAAGCGGAAGCTCAGGTACCCCCGGCTGCCCCTTGGTTGGGGACTTCTAGGGAGCATTGCCCCAGGGTGACCTCAGGGCACAGGAGGGGGACCAAGGAAAGCTCCTTGCTGGCCTGGGATGCCTCCTCTGGGTGAGCTGGGATGGCAGCCACTCAGGGCCGCTTCCCGCCTGGCAGGACCCGGGATGGAGCCAGGTCGCGCTGGAGCCCGTTTCCCCGCACCTGCCCCCACTCTACCCGCTGTTGAGCACCCAATCCCCTTCCTCCCCCACCCCTGTCCTGCCCTATTCCCCTCCTCCCCCACACCCTCGTCCCCTGTCTGTCTCCTTTCCTATCCTCCTCCTTCCCCGTGGCCCCTGGCTTCTGGCCTCCCTTCTCTGCCCACCCCTGGGTTTCTCCCTCTCCATCTGCAGCCTGGGCCTTGGCCTCTCCCTTGGAGGGCCTGGCCCTCTCTGGTCCTGGGCTCTCTCTTCCTCCATCTGTACGTCCGTTTGTCCATCTGCTTCTCTCTTTCTGTCTCAGTGCCCCCAACCACTCCAGCCCCTGGTCTCCTCCTCCCCTCCCACGGGCCTGTGCTGCCCCCTCCTGGCCGCAGTGCCTCACTGGCGTGGCCCTGGCCTGGTCATCAGGGCCCGGGGCCCAGCCCTCCCGTGCCTGGCACCGCAGCCCGGGTGCCGGGGTGGCCGAGATGGGGGACACGTGGTGGCCTAGATCTTGACACCACCCCCAACACACACAGGCCCTCCTCAGTGGGGACACACAGACAGACGCCACCTCTTTCTACGACCGCGTCTGGGCCGCCATCAGGGACAAGTACCGGTCCGAGGTCAGTGGAGGAGAAGGGAGGGGACTTGGTCCTCCCACCACACTGCCCCCACCCCGCCTCACAAAGGAGCTGCCTCTGGTTCTGGCACCTGGCCACCCTGGCCAGCTGGGTGGCCCCAGCACCCCCCACCGAGACCCCCCGACCCCAGCTGTCGGTCCTCCCTGCAGGCCCCGCAGGAGGGGCAGAGACACCGAGGCTGCCCCTTGGGCTGTGGTGTGAGCCTGAGGGTGTGGGTGGACCCTGAGTCCACAACACCGACCGCACTGCTCACCCGCCAGGCACTGCCCACCGACCTTCCAGCACCTTCTGCCCCTGACTTGACGGAGCCCAAGGAGGAGCAGCCGCCAGTGCCCTCGTCGCCCACAGAGGAGGAGGAGGAGGAGGAGGAGGAGGAGGAAGAAGAGGCTGAAGAAGAGGAGGAGGAGGAGGATTCCGAGGTGCAGGGGGAGCAGCCCAAGGTCCGTGTTTGGGGGAGAAGTGGAGACAGAGAGGGTGGGGGAAGGGCTACTCACTGACCCTGCCCCTGCCCCAGGAGGCCCCACCGCCACTGTCACCCCCGCAGCCGGCCAGCCCTGCTGAGGAAGACAAAATGCCGCCCTACGACGAGCAGACGCAGGCCTTCATCGATGGTGAGGGTGGGCGGGGGCCAGGCTCCTCGGGTGGGCCCAGCGTTTCCTGCCGTGGTGGCACAGGTCGAGGGAAGATCCTGAGCTTAGACCCTGCTCTGACTCGGCCAGCACAAGCCCCAGTATCTTGGGGAGTCCAGGAAGGGGGCCTAGGGTAAGCCAGTCCCACCCTCGCCAGCCCCAAGGGGCCCTTCTGCCTCCCCAAGGGCCGCAGCTTGTTTGTGTCACTCCTGGCCCCACTCGCTCAGGAGCTGGGAGCCTGGGCAGCAAGTCGGGGCTGCTCTATAGCTGGTGAGGCCCTCAAGGCTGTCGGGGTGAAGTCCTTGGCCAGAGCAAAATGAGGGTATGGGAGCACACAGCCACATCCATGGAACCCCGTTCCCCATCCTCCTGGATGGGGTTGAGGACATCTCTGACCTCCAACCCCTCTCCCAGCTGCCCAGGAGGCCCGCAACAAGTTCGAGGAGGCCGAGCGGTCGCTGAAGGACATGGAGGAGTCCATCAGGTAGCGGGGGCTGAGGAGCGGGGACACCTGTCCCACAGCGACTGCTCCTTGACTCCCAGGGGAGCTGGTGATGGGGAATCACTGAGGCAACCACAGGCTGGGCCTGGTCCCTGCAGGGAGGGTCCCTGGGAGGTGGCAGGGAGGACAGCCTGGGCACCATTGCTCAGCCAGACCCTCCTGTGTCTGTCGTCCTGGGTCAGGCACTGGCGTCCCCAGCTGCCCCTTAACCGCTCCGCCTCCCCTTCCAGGAACCTGGAGCAAGAGATTTCTTTTGACTTTGGCCCCAACGGGGAGTTTGCTTACCTGTACAGCCAGTGCTACGAGCTCACCACCAACGAGTGCGTCCCAGGAATGCAGGGGCCCCCACTGGCAGGGTGGGAGGCGGGTGGCCCCGGAAGTGGCACCGGCAGTTTCCTGATGGTTGGGGAACCATCTGCGGGTGGGGCCCGAGAGTGCTGCCTTCCATATTGAGGGGGAGCAGAAGCCAGGGGCCAGGTTTAGGGTTGGTCATTGGAGTTGGAGGTACCCTGTGTGTGGGGACTGGAGGAGGCGGTGGGGGGTGGCTGTGGGAGGAGGCTGGAATCCCTGCGTTCCCCAACCCATATGTCCCGGTCCTCCACAGATACGTCTACCGCCTCTGCCCCTTCAAGCTTGTCTCGCAGAAACCCAAACTCGGGGGCTCTCCCACCAGCCTTGGGTGAGTGGCTTGGGCTGGCCCCTTCCCTCTGCCTCCTCCTGGTGCCCCGACACCGGCCCAGCCCTCAGCACCCTGTGTCTCTCACAGCACCTGGGGCTCATGGATTGGCCCCGACCACGACAAGTTCAGTGCCATGAAGTATGAGCAAGGCACGGGCTGCTGGCAGGGCCCCAACCGCTCCACCACCGTGAGTGCCTGCAAGGCAGGGGAGCTGGGGCGGGGAGACCCAGGCCTGGCCCAGCCGAACCCTCTCGAGCACCCGTCTGCCCATCCCCAGGTGCGCCTCCTGTGCGGGAAAGAGACCATGGTGACCAGCACCACAGAGCCCAGTCGCTGCGAGTACCTCATGGAGCTGATGACGCCAGCCGCCTGCCCGGAGCCACCGCCTGAAGCACCCACCGAAGACGACCATGACGAGCTCTAGCTGGATGGGCGCAGAGGTGGGCGGGGAGGTGGAGTCTCGTCGGCCTGCCCCAGCAAGGGGAGGCGGCGGGCCCTGAGGAAGATGGACCCACATGGCCACTCTATCAACCTGTGTCCCCATGTTCCCTGCTTTTTTGTTTTGTTTTTTTGAGGTGGAGTCTCACTCTTTGGCCCAGGCTGGAGTGCAGTGATGCGACCTCAGCTGACTGCAACCTCTACCTCCCGGGTTCAAACAATTGTCTTGTCTCAGCCTCCCAAGTAGCTGGGATTACAGGTGTGCACCACCATGCCTGGCTAATTTTTAGTAGAGATGGGGTTTCACCATGTTGGCCTGGATGGTCTCGAACTCCTGACCTCAGATGATCCACCTGCCTTGGCCTCCCAAAGTGCTGGGATTACAGGCATGAGCCACCACACCCGGCCATCTCCTGCCTTTTTTTTTTTGGATGGAGTTTCACTCTTGTTGCCGAGGCTGGAATGTGGAATGCAATGGCGCGATCTCGGCTCACTGCAACCTCCACCTCCTGGGTGCAAGCAATTCTCCTGCCTCAGCCTCCTGAGTAATTGGGACTACAGGCGCCGCCACCACGCCTGGCTAATTTTTTTGTATTTTTAGTAGAGATGGGGTTTCCCTGTGTTAACCAGGTTGGTCTCGATCTCCTGACCTCGTGATCCGCCCTCCTCAGCCTCCCAAAGTGCTGGGATTACAGGCTTGAGCTACCATGTCTGGCCATCCCCTGCTTTTTAAAAAAAGATTTTTTTACTTTTGAAACTAAATAGGCCAGGCGTCGTGGCTCACATCTGTAATCCCAGCACTTTGGGAGGCTGAGGTGGGCGGATCACTTGAGGTCAGGAGGTTGAGACCAGCCTGACCAACATGGTGTAAAACCCCATCTCTACTAAAAATACAAAAAAATTAGCTGGGTGTGGTGGTAGGTGCCTGTAGTTCCAGCTGCTCAAGAGGCTGAAGCACAAGAATCACTTGAACCCAGGAGGTAGAGGTTGGAGTGAGCCAAGATCATGCTACTGCACTCTCCAGCCTGGGCTACAGAGTGAGACTCTCAAAAAAAAAAAATAATAATAATAAGAGAAAATAGACAAGGTCTCCAGGCTGGTCAACTCCTGGCCTCAAATGATCCTCCCACCTCAGCCTCCCAAGCAGCCGGGACTACAGGCAAACATCACCATGTCCAGCTGTCCCCAGCTTTCTAATCTGGTCTTTCTCTTGCCCCAGAACCTCAAGAAGGCATGAAGCCAGCCCCTGCAGTGCCGTCCACCCGCCCCTCTGGGCCTGCCTGTGGCTCTGTTGCCCTCCTCTGTGGCGGCAGGACCTTTGTGGGGCTTCGTGCCCTGCTCTGGGGCCCAGGCGGGGCTGGTCCACATTCCCAGGCCCCAACAGCCTTCAAAGATGGGTAAAGGAGCTTGCCCTCCCTGGGCCCCCCACCTTGGTGACTCGCCCCACCACCCCCAGCCCTGTCCCTGCCACCCCTCCTAGTGGGGACTAGTGAATGACTTGACCTGTGACCTCAATACAATAAATGTGATCCCCCACCCAAA
