# Supplementary material for: PRKCSH deficiency promotes an anti-tumor immune microenvironment via UPR activation and M1 macrophage polarization
Source: Cancer Cell Int. 2025 Dec 5;26:4. doi: 10.1186/s12935-025-04104-2 (PMC12797428; doi:10.1186/s12935-025-04104-2)
Supplement: Supplementary file 2 — Supplementary Material 2. [file 12935_2025_4104_MOESM2_ESM.zip › PRKCSH-KO sequencing/PRKCSH Knockout sequencing results.docx]

A549-WT Primer-F 152 Forward sequencing

TTTGACTCATCGTAGAAGTGATGATCTGCGGGGTACAGGAAGCTCAGCCCAGGGCAGGTAAGCGCCTTCTCCACCAAATACCACCTCTGTCCTCCCATCCCCACCAGCGGAGCACTACCGGGAAACTGAGTCAAAAGGCTGTAAATGCCTATCCCTAAGGCCCAATGTTGGCCCCTCCCACCTCCAGCCTGGCGGGAGGGTGAACAGGAGGACTCACTGGTGAGGGAGACGCCCCGGGGCCTCTTGACCTCCACGGCCCAGCACATGGGTAGCAGCAGCAGCAGCGGCAACAGCATCTCACCAGACGCTCTGAGGCCCGAAACGGGTTCTGTGGGAGGAAGCCTACAGCGGGGAGATGCGCTGTGTCTTCACGCTGCAGGAAGAAAGCGGATCCCGGTCAGTGGGAGAGGGCTACTTCCAGCCAATTGGATTCCTCCATCGCTTCCAGGCAATGAGGCCTCACTTTGTTTCCGATATGGGGGCGAGGGGCAGCGATCTCCCCAATCCTGGCCAGTCTCACTTTGCTCAAATCAATGGCGAGGATCTCAAGATCTCAAAACCTCCGCTCCCTGTGCCCAATTTACCCATTCCAAGCCCGCA

A549-WT Primer-R 782 Reverse sequencing

AACACCTGGGAGGGTAAGGAGCTCAGCCCAGGGCAGGTAAGCGCCTTCTCCACCAAATACCACCTCTGTCCTCCCATCCCCACCAGCGGAGCACTACCGGGAAACTGAGTCAAAAGGCTGTAAATGCCTATCCCTAAGGCCCAATGTTGGCCCCTCCCACCTCCAGCCTGGCGGGAGGGTGAACAGGAGGACTCACTGGTGAGGGAGACGCCCCGGGGCCTCTTGACCTCCACGGCCCAGCACATGGGTAGCAGCAGCAGCAGCGGCAACAGCATCTCACCAGACGCTCTGAGGCCCGAAACGGGTTCTGTGGGAGGAAGCCTACAGCGGGGAGATGCGCTGTGTCTTCACGCTGCAGGAAGAAAGCGGATCCCGGTCAGTGGGAGAGGGCTACTTCCAGCCAATTGGATTCCTCCATCGCTTCCAGGCAATGAGGCCTCACTTTGTTTCCGATATGGGGGCGAGGGGCAGCGATCTCCCCAATCCTGGCCAGTCTCACTTTGCTCAAATCAATGGCGAGGATCTCAAGATCTCAAAACCTCCGCTCCCTGTGCCCAATTGTGACCCAATTCCCGCCAACAGACCAAAGGGTTAAACAGCCTCAA

The above are the Sanger sequencing results of A549 wild-type cells, including both forward and reverse sequencing. Subsequently, PRKCSH-KO1, PRKCSH-KO2, and PRKCSH-KO3 were randomly sequenced in the forward or reverse direction respectively, and the sequencing results were obtained as follows. After sequence alignment, the base changes were obtained. Among them, when PRKCSH-KO1 is compared with the wild type, one base C is deleted, while PRKCSH-KO2 adds two bases CG. Both can obtain out-of-order results and achieve the purpose of knockout.

PRKCSH-KO1(1A8) Primer-R 782 Reverse sequencing

AATTAATCCTTGCGGGGGGTAAGGAGCTCAGCCCAGGGCAGGTAAGCGCCTTCTCCACCAAATACCACCTCTGTCCTCCCATCCCCACCAGCGGAGCACTACCGGGAAACTGAGTCAAAAGGCTGTAAATGCCTATCCCTAAGGCCCAATGTTGGCCCCTCCCACCTCCAGCCTGGCGGGAGGGTGAACAGGAGGACTCACTGGTGAGGGAGACGCCCCGGGGCCTCTTGACCTCCAGGCCCAGCACATGGGTAGCAGCAGCAGCAGCGGCAACAGCATCTCACCAGACGCTCTGAGGCCCGAAACGGGTTCTGTGGGAGGAAGCCTACAGCGGGGAGATGCGCTGTGTCTTCACGCTGCAGGAAGAAAGCGGATCCCGGTCAGTGGGAGAGGGCTACTTCCAGCCAATTGGATTCCTCCATCGCTTCCAGGCAATGAGGCCTCACTTTGTTTCCGATATGGGGGCGAGGGGCAGCGATCTCCCCAATCCTGGCCAGTCTCACTTTGCTCAAATCAATGGCGAGGATCTCAAGATCTCAAAACCTCCGCTCCCTGTGCCCAATTGTGACCCAATTCCCGCCAACAGACCAAAGGGATTAACAGCCTCCA


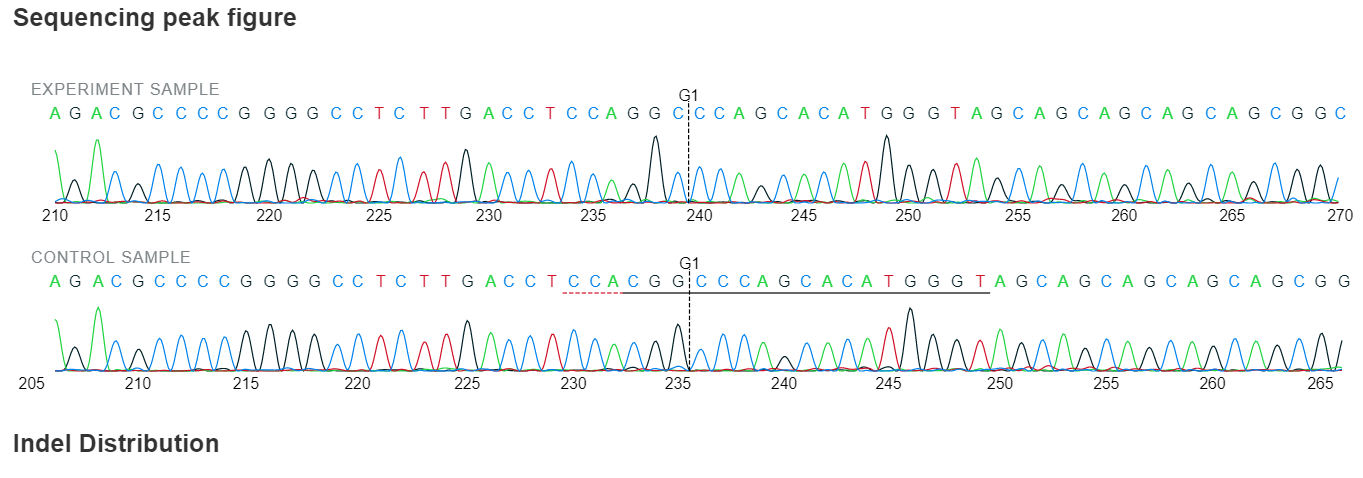


PRKCSH-KO2(2A11) Primer-F 152 Forward sequencing

TCAATTCGGGAAATTATTCAAATCAGGTGCAAGTTTAGCGGAGGTTTTGAGATCTTGAGATCCTCGCCATTGATTTGAGCAAAGTGAGACTGGCCAGGATTGGGGAGATCGCTGCCCCTCGCCCCCATATCGGAAACAAAGTGAGGCCTCATTGCCTGGAAGCGATGGAGGAATCCAATTGGCTGGAAGTAGCCCTCTCCCACTGACCGGGATCCGCTTTCTTCCTGCAGCGTGAAGACACAGCGCATCTCCCCGCTGTAGGCTTCCTCCCACAGAACCCGTTTCGGGCCTCAGAGCGTCTGGTGAGATGCTGTTGCCGCTGCTGCTGCTGCTACCCATGTGCTGGGCCGCGTGGAGGTCAAGAGGCCCCGGGGCGTCTCCCTCACCAGTGAGTCCTCCTGTTCACCCTCCCGCCAGGCTGGAGGTGGGAGGGGCCAACATTGGGCCTTAGGGATAGGCATTTACAGCCTTTTGACTCAGTTTCCCGGTAGTGCTCCGCTGGTGGGGATGGGAGGACAGAGGTGGTATTTGGTGGAGAAGGCGCTTACCTGCCCTGGGCTGAGCTTCCTGTACCCCGCAGATCATCACTTCTACGATGAGTCCAAGCCTTTC


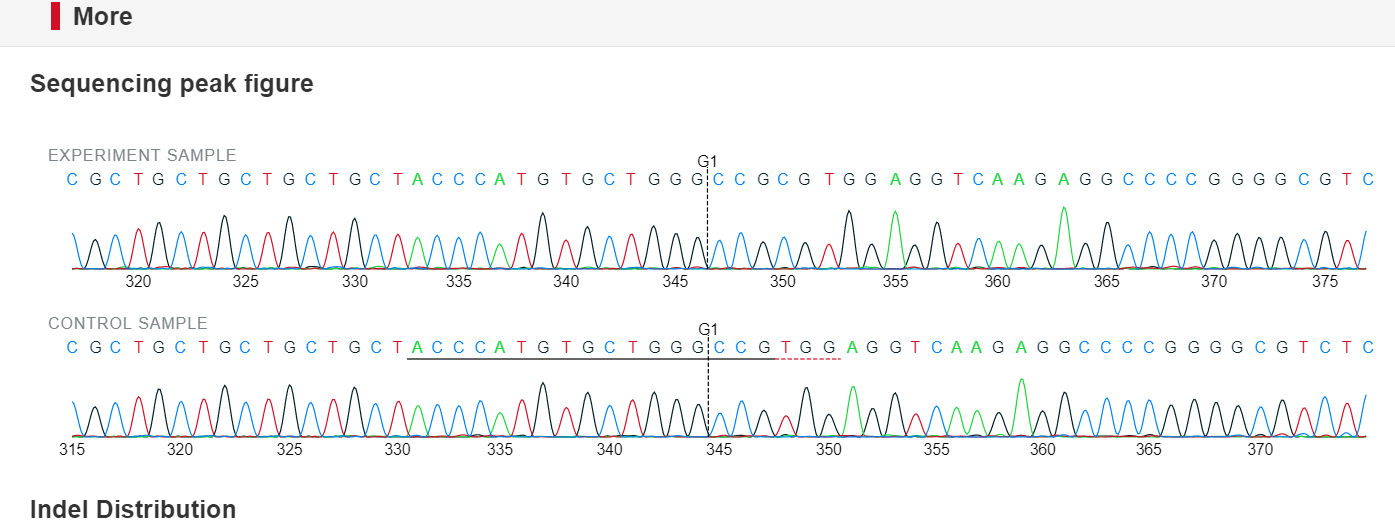


PRKCSH-KO3(X12D9) Primer-R 782 Reverse sequencing

GGAGGAATCTGCGGGTCGGAGCTCAGCCCAGGGCAGGTAAGCGCCTTCTCCACCAAATACCACCTCTGTCCTCCCATCCCCACCAGCGGAGCACTACCGGGAAACTGAGTCAAAAGGCTGTAAATGCCTATCCCTAAGGCCCAATGTTGGCCCCTCCCACCTCCAGCCTGGCGGGAGGGTGAACAGGAGGACTCACTGGTGAGGGAGACGCCCCGGGGCCTCTGGGACGAAGCCTACTGGGGGGAGATGCCCTGTGTCTTCACGCTGTCTGAAGAAAGCCTATCACGGCCAAAGGGAGAGGGGTGCTTCGAACCCTTTTGATTCCACATTCGCTTCGTCGTCATGATGCCTCAATATGTTTCATATATGGGGGCGAGAGACGGCTATCTCCCCACTCCTGGATTCCCTCACTTTGCTCAAATCAATGGGGAGGATCTTGTTATCTATAAACCTCCGATCCCTGTGCACCTCTGCGACCCTGTTCCCGCCCACTTAGCTCAGGGATTGACAGACTATCTCAAGATCTCAAAACCTCCGCTCCCTGTGCCCAATTGTGACCCAATTCCCGCCAACAGACCAAAGGGATTAAACAGCCTCA


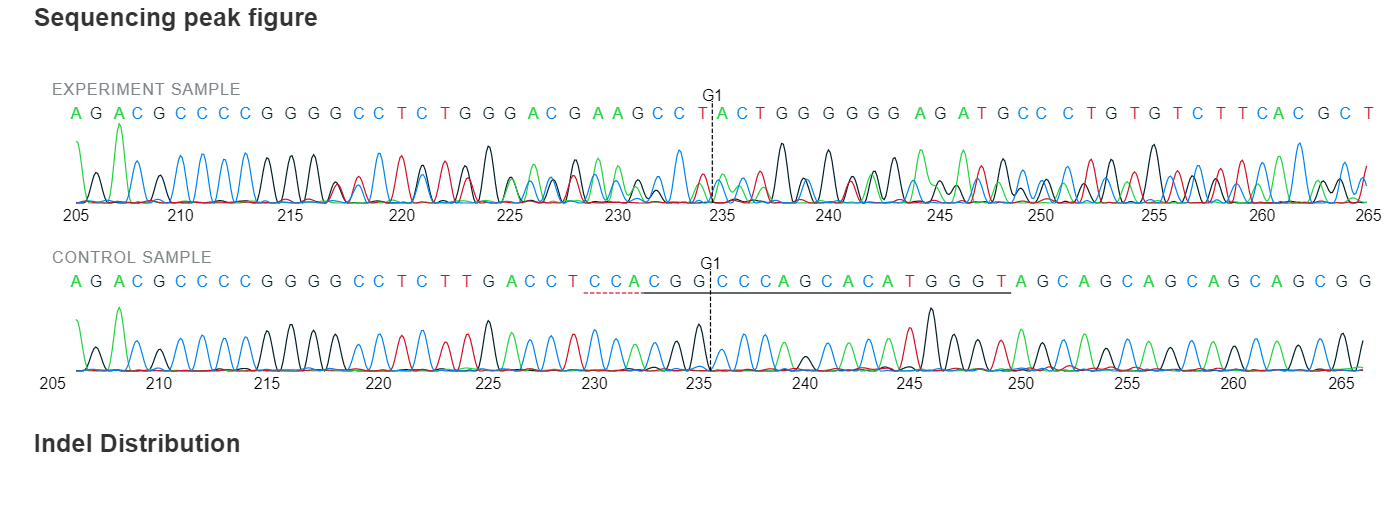


**PRKCSH Full length of the genome(FROM NCBI)**

**Primer-F 152：GAGGCTGTTAATCCCTTTGGTCT**

**Primer-R 782：TCTACGATGAGTCCAAGCCTTTC**

**gRNA ACCCATGTGCTGGGCCGTGG**

CTTTCTGCAGCAGGAACCGCGGCTGCTGGACAAGAGGGGTGCGGTGGATACTGACCTTTGCTCCGGCCTCGTGTAGGTGTGAACGAGCGGGTGGGAGGGCACCTCAGTTTCTTACAGGGGGCAACCGGAGGGTGCATGTGTGGGTGTGGACGGCGGAGGCTGTTAATCCCTTTGGTCTGTTGGCGGGAATTGGGTCACAATTGGGCACAGGGAGCGGAGGTTTTGAGATCTTGAGATCCTCGCCATTGATTTGAGCAAAGTGAGACTGGCCAGGATTGGGGAGATCGCTGCCCCTCGCCCCCATATCGGAAACAAAGTGAGGCCTCATTGCCTGGAAGCGATGGAGGAATCCAATTGGCTGGAAGTAGCCCTCTCCCACTGACCGGGATCCGCTTTCTTCCTGCAGCGTGAAGACACAGCGCATCTCCCCGCTGTAGGCTTCCTCCCACAGAACCCGTTTCGGGCCTCAGAGCGTCTGGTGAGATGCTGTTGCCGCTGCTGCTGCTGCTACCCATGTGCTGGGCCGTGGAGGTCAAGAGGCCCCGGGGCGTCTCCCTCACCAGTGAGTCCTCCTGTTCACCCTCCCGCCAGGCTGGAGGTGGGAGGGGCCAACATTGGGCCTTAGGGATAGGCATTTACAGCCTTTTGACTCAGTTTCCCGGTAGTGCTCCGCTGGTGGGGATGGGAGGACAGAGGTGGTATTTGGTGGAGAAGGCGCTTACCTGCCCTGGGCTGAGCTTCCTGTACCCCGCAGATCATCACTTCTACGATGAGTCCAAGCCTTTCACCTGCCTGGACGGTTCGGCCACCATCCCATTTGATCAGGTCAACGATGACTATTGCGACTGCAAAGATGGCTCTGACGAGCCAGGTGAGCCTTTTCTCTGTTCATCCATCAGATGTTTATTGAACACTGCTCAGTGCCAGGCCCTGTCTGTGTGACCAAGATACTACCTCTGCTGGCTCCCCTTTGAGTGGGCAGAAACAAGCTCAGAAGTCAGTGGTCAGTGTTATGGCCCCGGGCAGCTGGAGGAGCCCAGAGTCGTCCTCCTCCCAAGCCTCGGGGTCAGAGAAGGCTCCTCCAAGGAGGTGAGGTTTCAGTTGAATCCCGAAGGTGCTGCAGATGGAGGGGTCAACACGGGCAAACCCCTAGAAGTGGGAGACAGGGTGGGGCCAAGAGCAATCCAGCAGGAGACAGAGGTGAAGGGAGACCCTGGCTTTTTGTTTTTGTGTGTTTTGAAGAGACAGGGTCTTGCTTTGTTGCTGAGGCTGGAGTACAGTGGCATGATCGTAGCTCACTGCAGCCTGGAACTCTTGGGCTCAAGTCATCCTCCTGCCTTGGCCTCCCAAAGTGCTGGGGTTACAGGTGTGAGCCACCGTGCCTGGATGAGACCGTGTCTTTGGTTTTGTTTTTGTTTTGAGACGGAGTCTTGCTCTGTTTCCCAGGCTGGAGTGCAGTGGCGCCATCTCGGCTCACTGCAAGCTCCGCCTCCCGGGTTCACACCGTTCTCCTGCCTCAGCCTCCTGAGTAGCTGGGACTACAGGCACCCACCACCACGCCCAGCTAGTTTTTTTTTGTATTTTTAGTAGATACAGGGTTTCACTGTGTTAGCCAGGATGGTCTCGATCTCGTCACCTTGTGATCCACCCGCCTCGGCCTCCCAAAGTGTTGGGATTACAGGCGTGAGCCACCGTACCTGGCCTTGTTTTGTTTTGTTTTTAATTTTTTTTTTTGAGATGGAGTTTCACTCTTATCGCCCAGGCTGGAGTGCAGTGGCATGATCTTGGCTCACTGCAGTCTCCACCTCCCGGGTTCAAGCGATTCTCCTGCCTCAGCCTCCTGAGTAGCTGGGATTACAGGCACATGCCACCACATCCGGCTAATTTTTCGTATTTTTAGTAGAGATGGAGTTTCATCGTGTTGGCCAGGCTGGTCTCAAACTCATCACCTCAGGTGACCCACCCGCCTCGGCCTCCCAAAGTGCTGGGATTACAGGTGTGAGCCCCCGTGCCAGGTGGAGACCCTGTTTTGAAGGGCCTCAGTGAGAAGAGGCAGGAGGCGGCACTGGAGAGCTTTATTTCGAGCCCTGGCTGTGCAGTGTACAACTCTTTCCTTCCTTTGGTTTCCTTTCCTTTCTTGTGCTGTGTGCAGTGTGGGTCAGGGGCTCTTATCTGTGGATGGATGGGACATGTCTGTCCCTGAGCTGACTCCGAAAACCTTCCCTCCCTTCTTCCTCACAGGCACGGCTGCCTGTCCTAATGGCAGCTTCCACTGCACCAACACTGGCTATAAGCCCCTGTATATCCCCTCCAACCGGGTCAACGATGGTGTTTGTGGTAAGTGAAGATGCACCAGGATTCTGGAAAGGTGGTAGAGGGAGGGAGGGAGGAGGCACTGCCAGGTCTGATCTTGGCTTCTGCCTCTGCCACAGACTGCTGCGATGGAACAGACGAGTACAACAGCGGCGTCATCTGTGAGAACACCTGCAAGTACGTGGGTGACAGTACCCCTCCCATCACCCCACCCCAAGACTTTGCCTGGCTCCACCCAGTGAATCGGGCCCACTCTCTCTTCTGCTTTTCTGTATCGGGTTCTCTGTCTGTGCCAGGTGCCTTGTGAATCCTAATCCCCACCTTTCCCTCTAGTCTGGAAGCAGGTAGGATTGTTTCCCGATTCAACAGATGGGGGAACTCAAGTTTAGAAAGGGAAATGACTTCCCCAGGGTCACATAGGAGATAGCAGAGCCGGCACTTGGACTCTAAACTTTCCATTGTCACGCTCTGCAGCAGGTCCTGAAAACCGAAACACCCACAGGCAGGTAAAGTATACAACGGAAGGAGGCCAGGCGCGGTGGCTCACACCTGTAATCCCAGCACTTTGGGAGGCTGAGGCAGGCGGATCACGAGGTCAGGAGATCGAGACCATCCTGGCTGACATGGTGAAACTCCGTCTCACTAAAAATACAAAAAATTAGCTGGGTGTGGTGGCGGGCGCCTGTAGTCCCAGCTACTCGGTGGGCTGAGGCAGGTGAGTGGCGTGAACACGGGAGGCAGAGGTTGCAGTGAGCCGAGATCGCACCACTGCAATCCAGCCTGGGCAACAGAGTGAGACTCCGTCTCAAAAAAAAAGCAAAAAAAAAAAAAGCATACAACAGAAGGAGGCCAAGTATGAGGCAATACGGAGGGGTGGGGATTGTGGCATCTAGGGGAACAGCTGTTCTCAATTCCTGCCAGCTGATCCTTTTGTAGTTCATAGGCATGATGATTTATTTTTATTTATTTATATTTTTTGAGACAGGGTCTCACTCTGTCGCCCAGGCTGGAGTGCAGTGGCGTGATCTTGGCTCACTGCAACCTCTGCCCTTCGGGTTCAAGCAATTCTCACACCTCAGCCTCCCAAGTAGCTGGGAATACAGGCGTACCCCCACCACGCCTGGCTAACTTTTGTATTTTTGGTAGAGATGGTTTCACTATGTTGGCCAGGCTGGCCTCGAACGCCTGACCTCAGGTGATCTGCTCGCCTCAGCCTCCCAAAGTGCTAAGCCGCCGCGCTTGGCCACGATGATTAGGTTTTTATGCACGTGTGAGAGATACGCCTCCCTTAAACCTTGTTGCGACCTTGGCGCATTACCTGTCTGATATGAAAAAATAAGACCCGGCTCATGGTGGCTCCTGCCTGTAATCCCAGCATTTTGGGAGGCTGAGGCAGGAAGACTGCTTGAGCCCAGGAGTTTGAGACCAGCCTGGGCAAAATAGCGATACCCTATCTCTATAAAAAGTAAAAAAAAAAAATTAAAAAGTAAAAATAAAAATTGGAAATTATAATTTAAAAAATTACTGGTTGGGCATGTGGCTCACACCTGTAATCCCAGCACTTTGGGAGGCTAAGGCGAGAGGATCACCTTAACCCAGGAGTTTGAGACCAGCCTGGGCAACACAGTGAGACCCGTCTCAGAAAAATTTTTCTTTTCTTTTTTTTTTTTTTTTTTTTTTTTTTTGTGAGACAGAGTCTCACTCTGTTGCCCAGGCTGGATGGAGTGCAGTGGCGCGATCTCGGCTCACTCTAACCTCTGCCTCCCGGGTTCAAGCGATTCTTCTGCCTTAGCCTCCTGAGTAGCTGGGACTACAGGCATCCACCACCACACCCGGCTAAAATTTGTATTTTTAGTAGAGACGGTGTTTCACAATATTGGCCAGGCTGGTCTCGAACTCCTGACCTCATGATCCACCTGCCTTGGCCTCCCAAAGTGCGGGGATTACAGATGTGAGCCACCATGCCCGGCTGAAAATTTGTTAAAAAATTAGCCGGGTGTGTTGGTGCGTGTCTGTAGTCGCAGGTACTCAGGAGGCTGAGGCAGGAAGATTGCTTGGGCCTGGGAAGCAGAGGTTGCAGTGAGCTAAAAAGTAAAAAAAAAACAAAAAAAACACAAAGTCCTATCAGTTGTTGGCAGATGGTGTAAAACTCTCTGGATTCCACATCTTTTAAATAGAAGGTGGATTGAACACAACCAACACACTTCTTTTTTTTTTTGAGACGGAGTCTCACTCTGTCGCCCAGGCTGGAGTGCAGTGGTGTGATCTCCGCTCACTGCAAGCTCCGCTTCCCGAGTTCATGCCATTCTCCTGCCTCAGCCTCCCGAGTAGCTGGGACTACAGGCACCTGCCGCTACACCTGGCTAATTTTTTTTTTTGTATTTTTAGTAGAGATGGGGTTTCACCGTGTTAGCCCGGATGGTCTCGATCTCCTGACCTTGTGATCTGCCCGCCTCGGTCTCCCAAAGTGCTGGGATTACAGGTGTGAGCCACCGCCCCTGGCCCACACTTTTTTTTGAGTTAGAGTCTCGCTCTGTTGCCCAGGCTGGAGTGCAGTGGCATGATCTTTGCTTACCCCAACCTCCACATCCGGGTTCAAGTGATTCTCTTGCCTCAGCCTCCCGTGTAGCTGGGATTACACCGTGTAGCTGGGCGCCACCACGCCCAGCTAATTTTTGCATTTTCAGTAGAGACAGGGTTTCACCATCTTGGCCAGGCTGGTCTCGAACTCCTGACCTCAGGTGATCCGCCCGCCTTGGCCTCCCAAAGTGCTGGGATTACAGGCATGAGCCACCACGCCCAGCCAACCAACACACTTAGTAACCAGATTGTCTTTTTGTCTGGGAGTCATGATTTCTGTTCTGTCTTCTGTGGTTTTTTTATTTGTTTTAATTATGTGTAAAGACAGGGTCTTTATATGTTGCCCAGGCTGGTTGCGAACTCCTGGGCTCAAGTGATCCTCCTGCCTTGGCCTTTCAAACTGCTGGGATTACAGGCATGAACCACTGCACCCAGCTGATCTCCTGTCTCTTGAGAGTTGATATCTAGCTCCACCTCCTGGCACTCCCTAGTCCCTTCCCTGACCCCAGGGGCTTACAGAACCCACTGTTCCTCTTTCAAGGACAGGAATGAAGGATGCTGATGTTGAGAGAACCTGGGAGGAAGGCTTGCTCTCATCTTTCCCAGCATGGCTTGGTGGGGGGCCACAGCTGGATTGAGCTATTTTGGAAGAGGCAGACCTGGTGGATCCTAAGTGCCCCACTGGTGGTGCCTGTGTGTCTCCGCACCGCAGAGAGAAGGGCCGTAAGGAGAGAGAGTCCCTGCAGCAGATGGCCGAGGTCACCCGCGAAGGGTTCCGTCTGAAGAAGATCCTTATTGAGGACTGGAAGAAGGCACGGGAGGAGAAGCAGGTAAGGAACCCGCGGGGGCTGCCCCAGGGTGATCTGGGCCTTGAGGCCTCATAGTGTGGGCTTGCAAGGCTGGGGAGGGGTTTGCCCCTGACTGCTGTTCTGGGGCAAGTGACTGCCTTTCGGAGCTTTGCTTTCTCAGTTGTGTGCTAGGCGTTTAGTCATTGGCTAAGTATAATAGTTCCCGAGGGGGAGGCTGTCAGCTTAGGTGTTTATCAGGGCCTCGGGCATCAGACTAAAGCAGGCTGGAAGGGCCTCTCTGCCTCCTGGGGATGGGAGGGTTGCTTCTCTGTACCCCGGATCTGTGGTGCCATTGCCTGTAAGATGGGCTGATGACCAGATGACTCCCCAGTTCTGATGTTAGAAAGATAACGTCAGCCAGTAGCAGCTGGATTAATTCCATAGGGTGGGTGCTTTGGGGTCCCTAGGTGTGTGTCTCTCCCTGCCTTGCCTGTGTACTGGGCAGGAGGCTGGAAACCGACCTGGATTTGCCTCAAGGCATTCATTGCTAGTGGGAGGGACAGCCAGCCAGGGCTGCTGGACAAAGGGAAATTCGGGGTAGAGACTTCTTGCCCAGTTCCTCTCCCTCTCTGCCTTGGGGGCAGGAGGCAAGGAAGGCTTCTTGGAGGAGGAGGCCTCTATCTCACATGAAATCTGTTGGGTAATTTGGAGTTAGCCAGGTAGAGTGTTCCAGGCTGAAGGAACAGCATGTTTGAAGAACCGGAGGCAGAAGAGCGTTTAGGGGTCTGAATGTTGTCAGGGCGACTGGATCCTAGTAAGATAGGGATGGGGCAAATGGGGCTGGAGAATCAGGCAGGGGCCTGATTACCTGGGCCCTTGCGGGCTACATTGAGGCGTTTGGGCTTCTCTCCAGAGGGCTACAGGGAACCCCAGCTCGGGAGAGAGACCCAGCTTGGTGTGTGTTTTGGAACATCTCCCTGCTGCCCTGTGGAGTAGAGGCAGGGAGGTAGTAGTCAATGAGGAGGAGGCAGAACAGAGGAGAGCTGGTCTCTTGCCTTCTGCCCACCCAGAAAAAGCTCATTGAGCTACAGGCTGGGAAGAAGTCTCTGGAAGACCAGGTGGAGATGCTGCGGACAGTGAAGGAGGAAGCTGAGAAGCCAGAGAGAGAGGCCAAAGAGCAGCACCAGAAGCTGTGGGAAGGTATGGCAGAAATGGCCAAGGACTCACCTTCAGTCCTGGGTGGGAGCAGGTCTGGGCCTAGGAGTCTCCCGCTCATTATCTGTTGTCAGTTTCAGCAGGTTTTTGATCATGCTGCCCATCGCCTTGTGTGCTTTGAGGTTCGCTTGGATTGTGGCATATGTCAGGAGCCTCCTTTTTAAAAATTGTTTAAATAATTTGTTTTTTGAAACAGCGTCTCCCTCTGCCACCCGGGCTGGAGTACAGTGGCGTGATCTCAGCTCACTGCAACCTCTGCCTCCCAGGTTCAAGCAATTCTTGTGCCTCAGCCTCCTGAGTAGCTGAGATTACAGGCACCTGCCACCACATTTAACTAATTGTTTTGTATTTTTAGTAGAGACGGGGTTTCACAATGTTGGCCAGGCTGGTCCTGAACTCCTGACCTCAAGTGATCCACCTGCCTTGGCCCCCCAGAGTGCTGGGATTACAGGCATGAGCCACCTCGCCCGGCCGCCTACTTCCTTTTAACATCAATGTAGATTTTGTTATATGACTTTAGCAGGACTTAGTTAATCCAGTGACCATTTTTCCTATCAGGTGTTTGGTATTTGTCTTCACTAATTGTAAGAGCTTTTTTGTGGCTGGGCATGGTGGCTCACGCCTGTAATCCCAGCAGTTTGGGAGGCCGAGACGGGCAGATCACCTGAGGTCAGGAGTTCGAGACCACCCTGGCCAACATGGTGAAACGCCATCTCTACTAAAAGAAAAAATTATGGCCGGGCACAGTGGCTCATGCCTGTAATCCCAGCACTTTGGGAGGCTGAGGCAGGCAGATCACGAGGTCAGGAGATTGAGACCATCTTAGCCAACATGGTGAAACCCATCTCTACTAAAAATACAAAAATCAGCTGGACATGGTGGTGTGTGCCTGTAATCCCAGCTACTCGGGAGGCTAAGGCAGGAGAATTGCTTGAACCAGGAAGTTGGAGGTTTTCAGTGAGCTGAGATTGCGCCCCTATATTCCAGCCTGGCAACAGAGTGAGACTCCGTCTCAAAAAAAAAAAAAAAATTAGCCTGGCATGGTGGCATCTGCCTGTGGTCCCAGCTACTTGGGAGGCTGAGGCAGGAGAATCGCTGGAACCCGGGAGACAGAGGTTGCAGTGAGCTGAGATCTCGCCACTGCACTCCAGCCTGGGTGACAAAGCAATACTTCATCTAAAAAAAATAATAAAGAGCTTTTTCTATCTGGGGTTTGGCCCTTTGTCTGCAACTGAAGATTTTTTTTTAGTTTATTTATTTTTGAGATGGAGTCTCACTCTGTCGCCTAGGCTGAAGTGCCAATGGCCCGATCTCTGCTCACTGCAACCTCTACCTCCTGGGTTCAAGTGATTCTCCTGCCTAGGCTTCCTGAGTAGCTGGGATTACAGGCACCTGCCACCACAGCCGGCTAATTTTTGTAGTTTTAGTAGAGACGGGGTTTCACCGTGTTGGCCAGGCTGGTCTTGAACTCCTGACCTCAGGTGGCCTGCTTTGGCCTCCCAACGTGTTGGGATTACAGGCATGAGCCACGGTGCCCAGCTCTGTCTGCAACTGAAGATATTATTTCCCTTCTTCATTTGTTTTTGGTGCTAGAGCCTGGTGTTTTTTGGTAAGGCCACATTGCATGTAGAAAGGGAATTCACTGTCTGGCTCTGTCTGCCCCTGAATCTTTGTCGTGTTGCAAGCTTCCTTTCACTTGGGTCTGGGCAAGCTCAGGCCTGATTTTCCTCAGGATGTGTTCTCAGTGTGGAGCTGTGGAATCAGAGGGAAGCGCCATGTTTTGACCCAGATTCCCAAAGGGTGGCGGGGCTGAGCCAGGGCTCCGAGGGTAGATGGCCTGGATTGGGCCCCAGGCTACACCTCTCACTGGCTAGGTGATTATGGGCAGGTCCCTTCACCCACAGAAAGGTCAGGGACCCCTCAGGCCCTCATCTGTGAGACTCAGAACAGTGTCAGCCGGGTCTCCTGGGACATGCGGGATGTCCTGGGGTGACCTAAGACTCTCTAGAGGAAATACCAGCTGTTTTTATTCTGTTTGTAGAGGACCAAGCTGGAGCTTGGTATACAGTGGGAACTGAATCCTGGTATACAGTAGGAAGTTAATCATTGGAGCCGCTGTTGTGGCTGCTTTCCGCTCTGCTTAGGTTAGAGTAGCGACAGACAAAGGAGGGACTTTTGGGACAGAGGCTCGGTCCCCTTCTGTCCGGCTGGCCCCTCTTCCTGGGAGCAGCCCTGGGGACCCCCAAGATGCCCCCAGGACCGGTCTCCCTCCCTGTATCCTCCCCATCTCCCAGCATCCTTGGCCATGAAGTCTCATTCCTGCCAGCCCTGCCCCGACATGCCTCTGCCAGGTCCTAAACCTAAAGTCTCAGTGGTCGCTCTCCTCTCCCCAACCCCACCTCCCACCTCTGACCGTCTGCGCACTCCATCTGCGTCTCCCCAGAATCTGCCTCTGCTCCGTGCTTCTTCCTCAGTCCCCAGCCTGGGCCAGCCCTTGTTATCACCTGGCTTCTGCCCTCGCCCCCACCTCTGTCCCAACAGTGGCCTGACAGGGCTGCCCTCTGCCACCTGGGTTCCCACCTCCCTGCTCCAGCCCCACCAGTGGCCCTCCTGCCTCTGCACTCCCCACCTTGTCCCGGTCTCCCCCAACACAACCAGGCTTCAACCATTCACCTTGCTCTTCGTCTGTTCTCCCCTGGTAAGGGCAGGGACCTTTGTCTCCTCCACAGTCGTGTCCTCAGGGTCCAGCATGGGGCCAGGCATATAGTAGGCGCTTGGTGGCAAACGACCCTGTGGGGTGCGGGGGCTGATGGGAGCCGGTGGGTGGGCAGGGGGTGACAGAGGTGGCTTCTTTACAGAGCAGCTGGCTGCTGCCAAGGCCCAACAGGAGCAGGAGCTGGCGGCTGATGCCTTCAAGGAGCTGGATGATGACATGGACGGGACGTGAGTGTCCCCTAGTTGGAGCTGCCCACCTTTCCGTGGGCCTGGGTTTCCCTCCCCGCCACCCTCGCCTCTAGAAACCAGCCAGATCCTCCTTGGGTTCCCCCGGCGTGGGGTCCAGGCTGATCCCAAGCCCCGTGTGACCCCGCCTCTTACTCGTGGATGGCCAGGTCCAGGATGCCCTGGGCGAGGAGATAGGGGGACCACCCTCTCCCCAGGAGCTGGGCACAGACCCTCAGCCTCAGGGAGTGTGTCTAGGCATCTAGTGATCGGATGGCTTTTCCCAGCCCCACTACGCTCTGGGGAAAGCCAGACCTGGGTTCGAATCCTGGCTGTGCCTCTGAGCTTGCTGGGTTACCTGGTGGAGGGTGGACTTTTGGGGGCTGAGAGGCACACGGGGAGCCTAGGAGATGTCAGGTGCCAAGGAGCAGGGGGCTTCAGAGGGACCGAATGGGCAAGGGTGGGGGTGGGCTTTGCTGGGTGGGGGACCAAGGCAGATGGCAGCTTCAATGTGCCGGGTCCTGGGGTGGAAGGAGGTGCCACCTGGGTGAGTTGGTAGAGAGGTCATCGGACATTTGGGGGTCCAGGTATCTCCCAGTCCCACCCCTTACTTTGAGCTCCTTTGGGGTCATGGAGTCCTGAGGAGGGGGCAGGAGGGTGGGGAGGCCCCTACACCTTGAGGTCCTGAAGCAAGTTCCAGGGTGGGGCCCTGCAGGGAAGAACAGGTGGGCCACATGGTGCCCCCAACTGAGAGCCACTGGGGCCTCACCCCTCCAGTCTGCTTCTGCCACGCCCCCGCAGGGTCTCGGTGACTGAGCTGCAGACTCACCCGGAGCTGGACACAGATGGGGATGGGGCGTTGTCAGAAGCGGAAGCTCAGGTACCCCCGGCTGCCCCTTGGTTGGGGACTTCTAGGGAGCATTGCCCCAGGGTGACCTCAGGGCACAGGAGGGGGACCAAGGAAAGCTCCTTGCTGGCCTGGGATGCCTCCTCTGGGTGAGCTGGGATGGCAGCCACTCAGGGCCGCTTCCCGCCTGGCAGGACCCGGGATGGAGCCAGGTCGCGCTGGAGCCCGTTTCCCCGCACCTGCCCCCACTCTACCCGCTGTTGAGCACCCAATCCCCTTCCTCCCCCACCCCTGTCCTGCCCTATTCCCCTCCTCCCCCACACCCTCGTCCCCTGTCTGTCTCCTTTCCTATCCTCCTCCTTCCCCGTGGCCCCTGGCTTCTGGCCTCCCTTCTCTGCCCACCCCTGGGTTTCTCCCTCTCCATCTGCAGCCTGGGCCTTGGCCTCTCCCTTGGAGGGCCTGGCCCTCTCTGGTCCTGGGCTCTCTCTTCCTCCATCTGTACGTCCGTTTGTCCATCTGCTTCTCTCTTTCTGTCTCAGTGCCCCCAACCACTCCAGCCCCTGGTCTCCTCCTCCCCTCCCACGGGCCTGTGCTGCCCCCTCCTGGCCGCAGTGCCTCACTGGCGTGGCCCTGGCCTGGTCATCAGGGCCCGGGGCCCAGCCCTCCCGTGCCTGGCACCGCAGCCCGGGTGCCGGGGTGGCCGAGATGGGGGACACGTGGTGGCCTAGATCTTGACACCACCCCCAACACACACAGGCCCTCCTCAGTGGGGACACACAGACAGACGCCACCTCTTTCTACGACCGCGTCTGGGCCGCCATCAGGGACAAGTACCGGTCCGAGGTCAGTGGAGGAGAAGGGAGGGGACTTGGTCCTCCCACCACACTGCCCCCACCCCGCCTCACAAAGGAGCTGCCTCTGGTTCTGGCACCTGGCCACCCTGGCCAGCTGGGTGGCCCCAGCACCCCCCACCGAGACCCCCCGACCCCAGCTGTCGGTCCTCCCTGCAGGCCCCGCAGGAGGGGCAGAGACACCGAGGCTGCCCCTTGGGCTGTGGTGTGAGCCTGAGGGTGTGGGTGGACCCTGAGTCCACAACACCGACCGCACTGCTCACCCGCCAGGCACTGCCCACCGACCTTCCAGCACCTTCTGCCCCTGACTTGACGGAGCCCAAGGAGGAGCAGCCGCCAGTGCCCTCGTCGCCCACAGAGGAGGAGGAGGAGGAGGAGGAGGAGGAGGAAGAAGAGGCTGAAGAAGAGGAGGAGGAGGAGGATTCCGAGGTGCAGGGGGAGCAGCCCAAGGTCCGTGTTTGGGGGAGAAGTGGAGACAGAGAGGGTGGGGGAAGGGCTACTCACTGACCCTGCCCCTGCCCCAGGAGGCCCCACCGCCACTGTCACCCCCGCAGCCGGCCAGCCCTGCTGAGGAAGACAAAATGCCGCCCTACGACGAGCAGACGCAGGCCTTCATCGATGGTGAGGGTGGGCGGGGGCCAGGCTCCTCGGGTGGGCCCAGCGTTTCCTGCCGTGGTGGCACAGGTCGAGGGAAGATCCTGAGCTTAGACCCTGCTCTGACTCGGCCAGCACAAGCCCCAGTATCTTGGGGAGTCCAGGAAGGGGGCCTAGGGTAAGCCAGTCCCACCCTCGCCAGCCCCAAGGGGCCCTTCTGCCTCCCCAAGGGCCGCAGCTTGTTTGTGTCACTCCTGGCCCCACTCGCTCAGGAGCTGGGAGCCTGGGCAGCAAGTCGGGGCTGCTCTATAGCTGGTGAGGCCCTCAAGGCTGTCGGGGTGAAGTCCTTGGCCAGAGCAAAATGAGGGTATGGGAGCACACAGCCACATCCATGGAACCCCGTTCCCCATCCTCCTGGATGGGGTTGAGGACATCTCTGACCTCCAACCCCTCTCCCAGCTGCCCAGGAGGCCCGCAACAAGTTCGAGGAGGCCGAGCGGTCGCTGAAGGACATGGAGGAGTCCATCAGGTAGCGGGGGCTGAGGAGCGGGGACACCTGTCCCACAGCGACTGCTCCTTGACTCCCAGGGGAGCTGGTGATGGGGAATCACTGAGGCAACCACAGGCTGGGCCTGGTCCCTGCAGGGAGGGTCCCTGGGAGGTGGCAGGGAGGACAGCCTGGGCACCATTGCTCAGCCAGACCCTCCTGTGTCTGTCGTCCTGGGTCAGGCACTGGCGTCCCCAGCTGCCCCTTAACCGCTCCGCCTCCCCTTCCAGGAACCTGGAGCAAGAGATTTCTTTTGACTTTGGCCCCAACGGGGAGTTTGCTTACCTGTACAGCCAGTGCTACGAGCTCACCACCAACGAGTGCGTCCCAGGAATGCAGGGGCCCCCACTGGCAGGGTGGGAGGCGGGTGGCCCCGGAAGTGGCACCGGCAGTTTCCTGATGGTTGGGGAACCATCTGCGGGTGGGGCCCGAGAGTGCTGCCTTCCATATTGAGGGGGAGCAGAAGCCAGGGGCCAGGTTTAGGGTTGGTCATTGGAGTTGGAGGTACCCTGTGTGTGGGGACTGGAGGAGGCGGTGGGGGGTGGCTGTGGGAGGAGGCTGGAATCCCTGCGTTCCCCAACCCATATGTCCCGGTCCTCCACAGATACGTCTACCGCCTCTGCCCCTTCAAGCTTGTCTCGCAGAAACCCAAACTCGGGGGCTCTCCCACCAGCCTTGGGTGAGTGGCTTGGGCTGGCCCCTTCCCTCTGCCTCCTCCTGGTGCCCCGACACCGGCCCAGCCCTCAGCACCCTGTGTCTCTCACAGCACCTGGGGCTCATGGATTGGCCCCGACCACGACAAGTTCAGTGCCATGAAGTATGAGCAAGGCACGGGCTGCTGGCAGGGCCCCAACCGCTCCACCACCGTGAGTGCCTGCAAGGCAGGGGAGCTGGGGCGGGGAGACCCAGGCCTGGCCCAGCCGAACCCTCTCGAGCACCCGTCTGCCCATCCCCAGGTGCGCCTCCTGTGCGGGAAAGAGACCATGGTGACCAGCACCACAGAGCCCAGTCGCTGCGAGTACCTCATGGAGCTGATGACGCCAGCCGCCTGCCCGGAGCCACCGCCTGAAGCACCCACCGAAGACGACCATGACGAGCTCTAGCTGGATGGGCGCAGAGGTGGGCGGGGAGGTGGAGTCTCGTCGGCCTGCCCCAGCAAGGGGAGGCGGCGGGCCCTGAGGAAGATGGACCCACATGGCCACTCTATCAACCTGTGTCCCCATGTTCCCTGCTTTTTTGTTTTGTTTTTTTGAGGTGGAGTCTCACTCTTTGGCCCAGGCTGGAGTGCAGTGATGCGACCTCAGCTGACTGCAACCTCTACCTCCCGGGTTCAAACAATTGTCTTGTCTCAGCCTCCCAAGTAGCTGGGATTACAGGTGTGCACCACCATGCCTGGCTAATTTTTAGTAGAGATGGGGTTTCACCATGTTGGCCTGGATGGTCTCGAACTCCTGACCTCAGATGATCCACCTGCCTTGGCCTCCCAAAGTGCTGGGATTACAGGCATGAGCCACCACACCCGGCCATCTCCTGCCTTTTTTTTTTTGGATGGAGTTTCACTCTTGTTGCCGAGGCTGGAATGTGGAATGCAATGGCGCGATCTCGGCTCACTGCAACCTCCACCTCCTGGGTGCAAGCAATTCTCCTGCCTCAGCCTCCTGAGTAATTGGGACTACAGGCGCCGCCACCACGCCTGGCTAATTTTTTTGTATTTTTAGTAGAGATGGGGTTTCCCTGTGTTAACCAGGTTGGTCTCGATCTCCTGACCTCGTGATCCGCCCTCCTCAGCCTCCCAAAGTGCTGGGATTACAGGCTTGAGCTACCATGTCTGGCCATCCCCTGCTTTTTAAAAAAAGATTTTTTTACTTTTGAAACTAAATAGGCCAGGCGTCGTGGCTCACATCTGTAATCCCAGCACTTTGGGAGGCTGAGGTGGGCGGATCACTTGAGGTCAGGAGGTTGAGACCAGCCTGACCAACATGGTGTAAAACCCCATCTCTACTAAAAATACAAAAAAATTAGCTGGGTGTGGTGGTAGGTGCCTGTAGTTCCAGCTGCTCAAGAGGCTGAAGCACAAGAATCACTTGAACCCAGGAGGTAGAGGTTGGAGTGAGCCAAGATCATGCTACTGCACTCTCCAGCCTGGGCTACAGAGTGAGACTCTCAAAAAAAAAAAATAATAATAATAAGAGAAAATAGACAAGGTCTCCAGGCTGGTCAACTCCTGGCCTCAAATGATCCTCCCACCTCAGCCTCCCAAGCAGCCGGGACTACAGGCAAACATCACCATGTCCAGCTGTCCCCAGCTTTCTAATCTGGTCTTTCTCTTGCCCCAGAACCTCAAGAAGGCATGAAGCCAGCCCCTGCAGTGCCGTCCACCCGCCCCTCTGGGCCTGCCTGTGGCTCTGTTGCCCTCCTCTGTGGCGGCAGGACCTTTGTGGGGCTTCGTGCCCTGCTCTGGGGCCCAGGCGGGGCTGGTCCACATTCCCAGGCCCCAACAGCCTTCAAAGATGGGTAAAGGAGCTTGCCCTCCCTGGGCCCCCCACCTTGGTGACTCGCCCCACCACCCCCAGCCCTGTCCCTGCCACCCCTCCTAGTGGGGACTAGTGAATGACTTGACCTGTGACCTCAATACAATAAATGTGATCCCCCACCCAAA
